# Let-7 regulates cell cycle dynamics in the developing cerebral cortex and retina

**DOI:** 10.1038/s41598-019-51703-x

**Published:** 2019-10-25

**Authors:** Corinne L. A. Fairchild, Simranjeet K. Cheema, Joanna Wong, Keiko Hino, Sergi Simó, Anna La Torre

**Affiliations:** Department of Cell Biology and Human Anatomy, University of California - Davis, Davis, CA USA

**Keywords:** Cell proliferation, Developmental neurogenesis

## Abstract

In the neural progenitors of the developing central nervous system (CNS), cell proliferation is tightly controlled and coordinated with cell fate decisions. Progenitors divide rapidly during early development and their cell cycle lengthens progressively as development advances to eventually give rise to a tissue of the correct size and cellular composition. However, our understanding of the molecules linking cell cycle progression to developmental time is incomplete. Here, we show that the microRNA (miRNA) let-7 accumulates in neural progenitors over time throughout the developing CNS. Intriguingly, we find that the level and activity of let-7 oscillate as neural progenitors progress through the cell cycle by *in situ* hybridization and fluorescent miRNA sensor analyses. We also show that let-7 mediates cell cycle dynamics: increasing the level of let-7 promotes cell cycle exit and lengthens the S/G2 phase of the cell cycle, while let-7 knock down shortens the cell cycle in neural progenitors. Together, our findings suggest that let-7 may link cell proliferation to developmental time and regulate the progressive cell cycle lengthening that occurs during development.

## Introduction

Cell cycle progression and cell cycle exit are tightly regulated in neural progenitors during central nervous system (CNS) development^1–3^. Generally, early development of the CNS is characterized by rapid cell divisions but, as developmental time progresses, the length of the neural progenitor cell cycle increases as more progenitor cells simultaneously undergo asymmetric divisions and differentiate^4–10^. The correct balance of continued proliferation and cell cycle exit is required to establish the proper size of the adult tissue. Furthermore, neurons and glia are produced in a stereotypic birth order that is conserved across species, such that some cell populations are made only at early stages of development (early-born) while other neurons and glia are generated at later ontogenic stages (late-born)^11,12^. As a result, the timing at which cells exit the cell cycle affects their final cell fate^1,13,14^, underscoring the importance of cell cycle control on ensuring neuronal diversity in the adult.

A growing body of research indicates that neural progenitors pass through waves of competence to both acquire and lose the ability to make specific cell types^11,15^. It has further been shown that an “intrinsic clock” provides progenitor cells with a sense of temporal identity^16–20^. However, the exact molecular mechanism(s) responsible for coupling competence with proliferation are not known.

Increasing evidence has shown that microRNAs (miRNAs) mediate developmental timing in many regions of the CNS^21–25^. MiRNAs are small (20–24 nucleotide), single-stranded RNA molecules that bind to target mRNAs in an antisense fashion and negatively regulate protein levels^26^. In the mammalian neocortex and retina, mature miRNAs are required for driving temporal shifts in progenitor competence, such that loss of the miRNA-processing enzyme Dicer leads to an extensive increase in the number of early-born cell fates generated at the expense of late-born cells^22,27,28^. The identities of the specific miRNAs involved in regulating developmental timing in the retina have recently been elucidated, and include: let-7, miR-9 and miR-125b^29^. In the retina, these three miRNAs accumulate as development progresses^29^, making them excellent candidates for controlling several of the cellular changes that occur during developmental time.

Let-7 was one of the first miRNAs identified as a member of the heterochronic pathway that drives developmental transitions in the nematode *C*. *elegans*^26,30,31^. It has since been implicated in the regulation of developmental timing in a wide array of species, including mammals, and it is also involved in cancer progression and stem cell differentiation^32–36^. In different contexts, let-7 has been shown to directly target multiple components of the basic cell cycle machinery, including: Cyclin D1, Cyclin D2, CDK4, CDK6 and CDC25A^37–39^. In the developing CNS, let-7 has been shown to drive neural differentiation by repressing genes that promote proliferation and cell cycle progression. This is true both in the embryonic retina, where let-7 has been shown to repress the epigenetic regulator Hmga2^40^, and in the cortex, where it targets Hmga2^41^ and the proliferation-promoting, nuclear receptor TLX^42,43^. Furthermore, downregulation of let-7 is essential for retinal regeneration in adult zebrafish retinas^44^, and its loss in adult rodent retinas, which do not spontaneously regenerate, promotes Müller glia cell cycle re-entry and de-differentiation^45–47^.

Despite all the roles described for let-7 in CNS development, a comprehensive characterization of its expression pattern is still missing. In the present study, we characterize the expression pattern of let-7 in the developing mouse CNS by *in situ* hybridization. We find that the level of let-7 in neural progenitors increases over time throughout the developing CNS. The spatial resolution provided by our *in situ* hybridization assays allowed us to observe variations in let-7 levels within the cortical ventricular zone (VZ) progenitor population that correlate with the location of progenitor cells as they undergo cell divisions and move via interkinetic nuclear migrations. We use *in vitro* miRNA sensor assays to confirm that let-7 activity oscillates as cells undergo cell cycle. Importantly, we find that experimentally manipulating let-7 levels in multiple models of neural progenitors impacts cell cycle kinetics. Consistent with the current literature, we find that let-7 promotes cell cycle exit; however, we provide novel evidence that let-7 also controls the length of the neural progenitor cell cycle. Using the Fluorescence Ubiquitination-based Cell Cycle Indicator (FUCCI), we show that let-7 regulates the cycle during S/G2. Together, our findings suggest that let-7 is regulated during the cell cycle, and that let-7 simultaneously regulates cell cycle dynamics. Furthermore, our data support the hypothesis that let-7 is one component of an “intrinsic clock” mechanism that links proliferation to developmental time.

## Results

### Let-7d expression in the developing central nervous system

**Figure 1:**
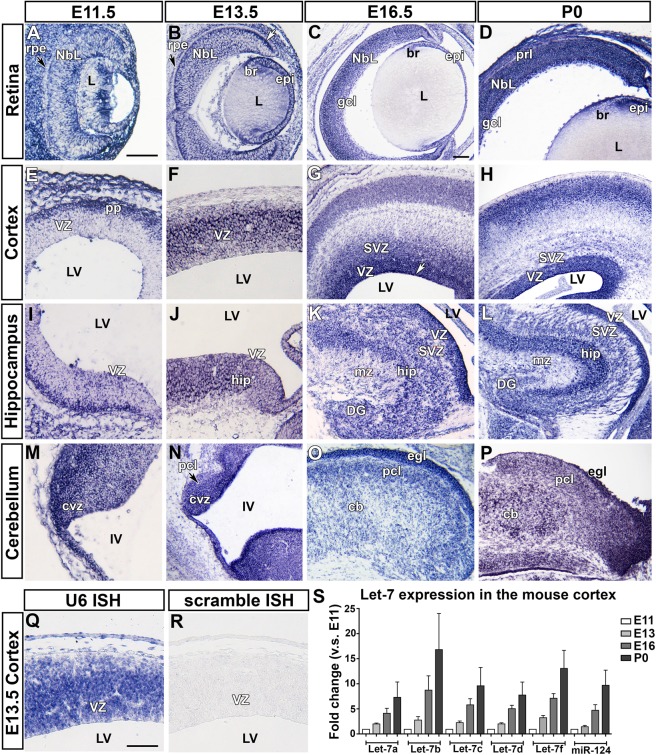
Let-7d expression in the embryonic mouse central nervous system. Let-7d expression pattern in the embryonic central nervous system analyzed using *in situ* hybridization and qRT-PCR. (**A**–**D**) Sagittal sections of the mouse retina at E11.5 (**A**), E13.5 (**B**), E16.5 (**C**) and P0 (**D**). Let-7d is absent from the retinal pigment epithelium (black arrowheads in **A**,**B**) and high along the apical surface (white arrowhead in **B**). (**E**–**H**) Horizontal sections of the mouse cerebral cortex at E11.5 (**E**), E13.5 (**F**), E16.5 (**G**) and P0 (**H**). From E16.5 onwards, the level of let-7d is particularly high along the apical surface of the lateral ventricle (white arrowhead in **G**). **(I**–**L)** Horizontal sections of the mouse hippocampus at E11.5 (**I**), E13.5 (**J**), E16.5 (**K**) and P0 (**L**). (**M**–**P**) Horizontal sections of the mouse cerebellum at E11.5 (**M**), E13.5 (**N**), E16.5 (**O**) and P0 (**P**). **(Q**,**R)** *In situ* hybridization using the following control probes: U6 positive control (**Q**) and scrambled negative control (**R**) on a horizontal section of the mouse cortex at E13.5. **(S)** qRT-PCR for let-7a,b,c,d,f and miR-124 on samples of mouse cortex collected at E11, E13, E16 and P0. All let-7 family members, and miR-124, increase over developmental time. Images shown in (**A**,**B**,**E**,**F**,**I**,**J**,**M**,**N**,**Q**,**R**) were taken at 20 × (scale bar in A = 100 µm). Images shown in (**C**,**D**,**G**,**H**,**K**,**L**,**O**,**P**) were taken at 10 × (scale bar in C = 100 µm). Rpe, retinal pigment epithelium; Nbl, neuroblastic layer; L, lens; br, bow region of the lens; epi, epithelium of the lens; gcl, ganglion cell layer; prl, photoreceptor layer; VZ, ventricular zone; LV, lateral ventricle; SVZ, subventricular zone; hip, hippocampus; mz, marginal zone; DG, dentate gyrus; CVZ, cerebellar ventricular zone; IV, fourth ventricle; pcl, Purkinje cell layer; cb; cerebellum; ecl, external germinal layer.

To determine the spatiotemporal expression pattern of let-7 in the developing CNS, we performed miRNA *in situ* hybridization on embryonic mouse tissue samples ranging from embryonic day (E) 11.5 to postnatal day (P) 0 (birth) using a locked nucleic acid (LNA) detection probe against let-7d. We found that let-7d was widely and dynamically expressed throughout the CNS (Table 1). In the retina at E11.5, let-7d levels were low in the progenitor cells (Neuroblastic layer (NbL); Fig. 1A). From E13.5 to P0 (Fig. 1B–D), all retinal layers had detectable levels of let-7d, but let-7 was slightly enriched in the NbL and highest along the apical surface (see white arrow in Fig. 1B). In the lens, let-7d was absent from the lens fibers, but high in the lens epithelium and bow region (Fig. 1B–D). Let-7d was notably absent from the retinal pigment epithelium at E11.5 and E13.5 (Fig. 1A,B, Table 1) but expressed in this tissue from E16. Similarly, as the patterning of the retina takes place, the ciliary body exhibited the highest levels of let-7, and let-7 expression remained high at all the ages tested. Similarly, in the neocortex at E11.5, let-7d levels were initially low in ventricular zone (VZ) progenitors and high in post-mitotic neurons of the preplate (pp; Fig. 1E). However, at E13.5, the pattern of let-7d was reversed; at this stage let-7d levels were enriched in VZ progenitors and lower in post-mitotic neurons (Fig. 1F). Let7-d level continued to increase in VZ progenitors at E16.5 and P0 (Fig. 1G,H, Table 1), and was highest apically, near the lateral ventricle (LV; white arrow, Fig. 1G). A similar pattern was observed in the hippocampus and cerebellum (Fig. 1I–P, Table 1), with early VZ progenitors initially containing low levels of let-7d at E11.5 and increasingly higher levels from E13.5 onward.

**Table 1:** Let-7d expression in the embryonic mouse central nervous system.

Olfactory bulb	E11.5	E13.5	E16.5	P0	
**Telencephalon**					
Olfactory ventricle	+/−	++	+++		*
Glomerular cell layer			+	+	
Mitral cell layer			++++	++++	
Granular cell layer				++	
**Neocortex**					
Marginal zone	+++	−	−	−	
Cortical plate		−/+			
Intermediate zone		−	−/+		
Subplate		+	+		
Preplate	+++				
Ventricular zone	+	++	+++	++++	*
Subventricular zone/RMS		+	++	++++	*
II-III layer				+++	
IV-V-VI layer			++	++	
Cingulate/Retrosp.	+	++	+++	+++	
Entorhinal cortex		+	+	+++	
Piriform cortex	+	+++	+++	++++	
Hippocampus					
Primordium (ventricular zone)	+	++	+++	+++	*
CA1/CA2		+	+	++	
CA3			+	++	
Dentate Gyrus				+	
Fimbria/fornix	+/−	+/−	+/−	+/−	
Subiculum		++	+	+	
**Basal forebrain**					
Ganglionic eminence	++	+++	+++		*
Caudate/Putamen		+	+	++	
Globus Pallidus		−	+	+	
Accumbens nucleus		++	+++	+++	
Medial septum		+	++	+	
Dorso-lateral septum		+	+	++	
Amygdaloid complex	++	++	++++	++++	
**Diencephalon**					
Epithalamus/habenula	+	++	++	++	
Thalamus					
Dorsal	++	++	++	++	
Medial	+	++	+++	+++	
Lateral	+	+	+	+	
Lateral geniculate nuclei	++	+	++	++	
**Hypothalamus**					
Anterior and preoptic nuclei	+	++	++	++	
Dorso-medial nuclei	++	++	++	++	
Arcuate	++	++	++	+	
Mammillary nuclei	+	+	+	+/−	
**Retina and eye**					
Neural retina					
Neuroblastic layer	+	++	+++	+++	*
Outer nuclear layer				++	
**Inner nuclear layer**					
Ganglion cell layer		++	++	++	
Optic stalk/optic nerve	+	+/−	−	−	
Ciliary body			++	+++	
Retinal pigment epithelium	+/−	−	++	++	
Lens epithelium	++	+++	+++	+++	
1ary lens fibers	+++	+	−	−	
Bow region	++	+	+/−	+/−	
Corneal epithelium	+	−/+			
Cornea			+	+	
**Mesencephalon**					
Periaqueductal gray area		+	+	+	
Dorsal raphe		+	+	+	
Superior colliculus		+++	+++	+++	
Inferior colliculus		++	++	++	
**Rhombencephalon**					
Cerebellum					
Upper rhombic lip	+	+++	++++	++++	*
Purkinje layer		++	+++	+++	
External germinative layer		+	+++	++++	*
Vermis			+	+	
**Primer List**					
Primer Name	Primer Sequence				
5s RT	ACTGCTGCGCTGCAGGGTCCGAGGTATTCGGCGCAGCAGTAAAGCCTA				
Let-7a/f RT	CGCTACACGCTTGCGTGTTATTTCCTGATGGCGTGTAGCGAACTATACA				
Let-7b RT	ATCAGGTAGCGCACGCTAGGGAACATCAGGGCTACCTGATAACCACAC				
Let-7c RT	TCGCGACTGCCAGTTCAGCAGGGTCATAGGGCAGTCGCGAAACCATAC				
Let-7d RT	TCGCGACTGCCAGTTCAGCAGGGTCATAGGGCAGTCGCGAAACTATGC				
miR-124 RT	TGTATCTTGCGGATGACTCAACGCGGGCTAGCAAGATACAGGCATTCA				
5s Forward	GCCATACCACCCTGAACG				
5s Reverse	TGCAGGGTCCGAGGTATTCG				
Let-7a Forward	CGGCCTGAGGTAGTAGGTTG				
Let-7a Reverse	GCTTGCGTGTTATTTCCTGATGG				
Let-7b Forward	CGCCCTGAGGTAGTAGGTTG				
Let-7b Reverse	GCACGCTAGGGAACATCAGG				
Let-7c Forward	CGCGGGTGAGGTAGTAGGTT				
Let-7c Reverse	CAGTTCAGCAGGGTCATAGG				
Let-7d Forward	CGCGGGAGAGGTAGTAGGTT				
Let-7d Reverse	CAGTTCAGCAGGGTCATAGG				
Let-7f Forward	CGCCGCTGAGGTAGTAGATTG				
Let-7f Reverse	GCTTGCGTGTTATTTCCTGATGG				
miR-124 Forward	CCGTTAAGGCACGCGGTGA				
miR-124 Reverse	GGATGACTCAACGCGGGCTA				

Let-7d expression pattern in the embryonic mouse central nervous system at E11.5, E13.5, E16.5 and P0 analyzed using *in situ* hybridization. Very low (+/−), low (+), moderate (++), high (+++), and very high (++++) levels of Let7d expression. No detectable expression is indicated (−). An empty cell means that the corresponding areas were not defined at that developmental stage. * Indicates a region containing neural progenitors.

We performed a number of control *in situ* hybridizations to validate our let-7d *in situ* profile. First, we used a control LNA probe against the small nuclear RNA U6 (Fig. 1Q), and found that it evenly labelled the nuclei of all the cells, while a negative control, an LNA miRNA scrambled probe, suggested there was little non-specific background staining (Fig. 1R). We also performed *in situ* hybridization analyses on Emx1-Cre;Dicer^F/F^ mutants at E13.5, in which Dicer is conditionally deleted in the dorsal telencephalon from E9.5^28,48^. As expected, let-7d expression was very low in the cortex of  Emx1-Cre;Dicer^F/F^ mutant, compared to wild-type littermates but no difference was observed in the retina and other diencephalic structures, where the recombinase Cre is not expressed (Fig. S1A–D). Lastly, we performed a control *in situ* hybridization for miR-183 and found that it was expressed specifically in photoreceptors after P3 (Fig. S1E–G), consistent with previous studies^49^ (Fig. S1).

To assess the relative expression of other let-7 family members in the developing CNS, we performed *in situ* hybridization using an LNA probe for let-7c and quantitative RT-PCR (qPCR) for let-7a, 7b, 7c, 7d, and 7 f. The expression pattern of let-7c was similar to that of let-7d in both the retina and cortex at E13.5 (Fig. S1H,I). Furthermore, a recently published *in situ* expression profile for let-7b and 7c is consistent with the let-7d expression profile we show here at later stages of development^41^. A thorough expression profile for let-7 family members in the developing retina has already been described^29^, and we found that likewise, in the cortex, all let-7 family members assessed increased with developmental time (Fig. 1S). As expected, miR-124 also increased between E11.5 and P0, which is consistent with current literature showing that miR-124 is upregulated upon neuronal differentiation in cell culture^50^ and over developmental time in the cortex by *in situ* hybridization^41^.

### Let-7 levels oscillate when neuronal progenitors undergo cell divisions

**Figure 2:**
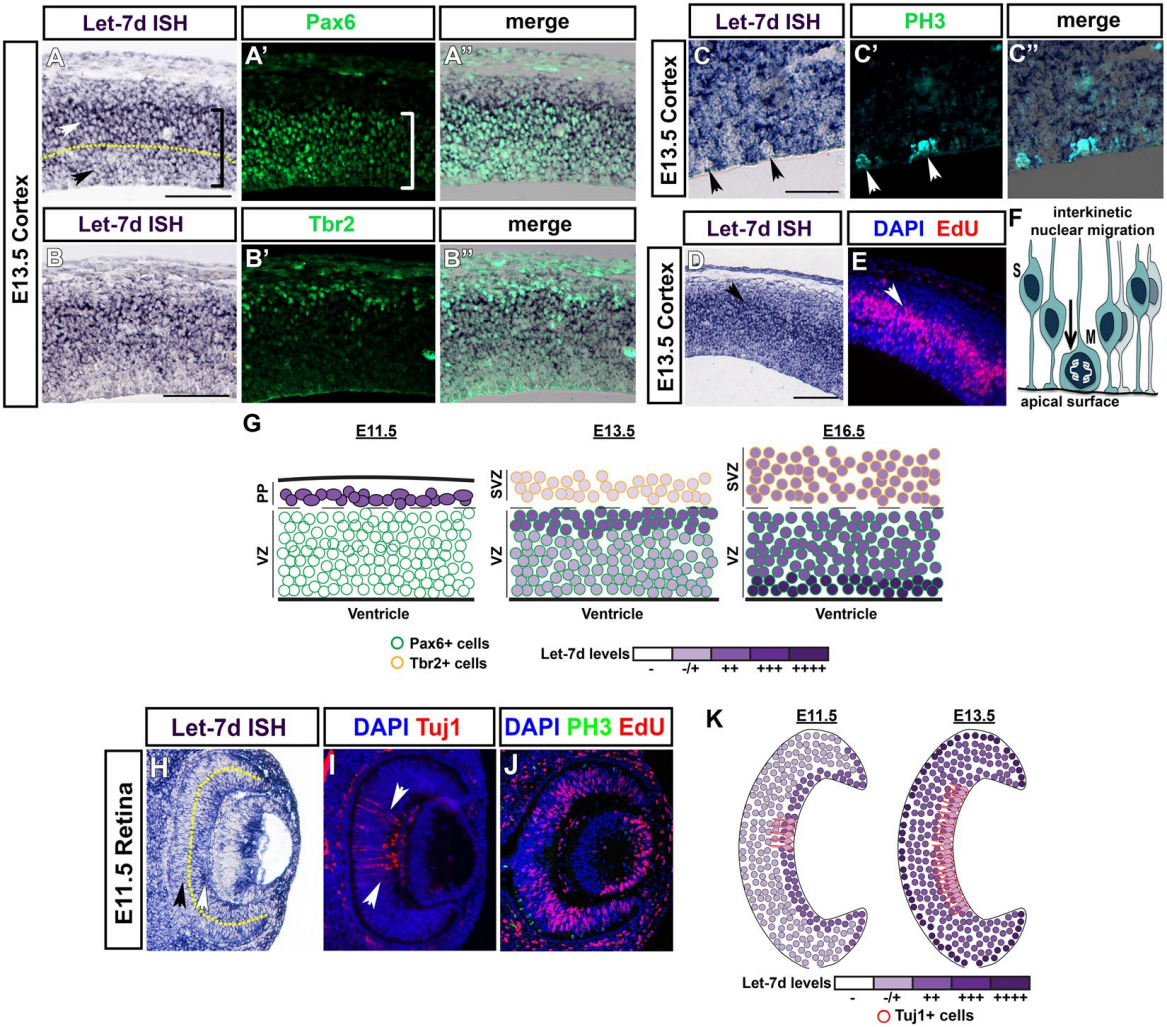
Let-7d levels oscillate when cells undergo cell divisions. (**A**–**E**) A side-by-side comparison of horizontal sections from mouse cortex, at E13.5, analyzed by *in situ* hybridization for let-7d and with immunostaining for progenitor and cell cycle markers. (**A**,**B**) *In situ* hybridization for let-7d (purple) and immunostaining for Pax6 (green in A’) or Tbr2 (green in B’). Merged images in A” and B”. Let-7 levels vary (compare above and below yellow dashed line in A; arrowheads) within the Pax6+ domain (brackets in **A**,**B**). (**C**) *In situ* hybridization for let-7d (purple) and immunostaining for PH3 (green in C’). Merged image in C”. Let-7d levels are low in PH3+ cells (arrowheads). (**D**,**E**) *In situ* hybridization for let-7d (purple in **D**) shows that cells with the highest level of let-7d reside in the region where EdU+ cells in S-phase are present (red in E; see arrowheads). DAPI nuclear staining shown in blue (**E**). **(F)** Schematic of interkinetic nuclear migration. Progenitors migrate to the apical surface to divide. **(G)** Schematic highlighting the dynamic changes in let-7d expression pattern over time in the mouse cortex. **(H**–**J)** *In situ* hybridization for let-7d in the retina at E11.5 (purple in **H**) showed that let-7d levels vary within retinal progenitors (compare arrowheads on either side of yellow dashed line in **H**). Immunostaining for Tuj1 (red in **I**) and staining with DAPI (blue in **I**) showed that fluctuations in let-7d levels do not correlate with newly-generated, Tuj1+, post-mitotic cells (white arrowheads). (**J**) EdU+ cells in S-phase (red), immunostaining for PH3 (green), and DAPI nuclear staining (blue) suggest that cells having a high level of let-7d reside in the vicinity of EdU+ cells. **(K)** Schematic highlighting the dynamic changes in let-7d expression pattern over time in the mouse retina.

One striking observation from our *in situ* hybridization profile was that let-7d levels varied within the cortical progenitor population in the VZ at E13.5. We first hypothesized that the higher level of let-7d might correspond with intermediate progenitors, which populate the more basally-located subventricular zone. To investigate this possibility, post-hybridization cortical sections were immunostained for the intermediate progenitor marker Tbr2. We found that Tbr2+ cells did not colocalize with the highest levels of let-7d but instead all intermediate progenitors had low levels of let-7d (Fig. 2B-B”). However, when we stained the samples for the VZ marker Pax6, we found that the levels of let-7d were variable within the VZ Pax6+ progenitors. The Pax6+ population spans the majority of the cortex at this age (Fig. 2A’); however, let-7d levels varied along the apical-basal axis of this population, with progenitors in the basal region having a higher level of let-7d as compared to those located more apically (Fig. 2A; compare white arrowhead above with black arrowhead below the yellow line).

Interkinetic nuclear migration (IKNM), during which progenitor cell bodies move towards the apical surface to undergo cell divisions, is a hallmark of neuronal progenitor behavior^51–54^ (Fig. 2F). Thus, we reasoned that the apical region in the cortex, where we observed low levels of let-7d, might correspond to progenitors actively undergoing cell divisions. To test this hypothesis, we immunostained for phosphohistone H3 (PH3), which stains condensed chromatin in late G2 and mitosis, following let-7d *in situ* hybridization. As expected, PH3+ cells were located apically, directly adjacent to the lateral ventricle (Fig. 2C’) and, interestingly, PH3+ cells often had lower levels of let-7d compared to neighboring cells (Fig. 2C,C”; quantification in Fig. S2A), suggesting that let-7d levels are low in cortical progenitors undergoing mitosis at this developmental stage. Additionally, providing a pulse of the thymidine analog 5-ethynyl-2’-deoxyuridine (EdU) 90 min prior to embryo collection at E13.5 allowed us to visualize cortical progenitors in S-phase. The cortical progenitors with the highest level of let-7d by *in situ* resided in the same region where EdU+, S-phase progenitors were located (Fig. 2D,E). Interestingly, the lower level of let-7d in dividing cells was a temporal phenomenon, and by E16.5 cortical progenitors apically located exhibit notably high levels of let-7d (Figs 1G,H and 2G).

A similarly dynamic pattern was observed in the retina at E11.5 (Fig. 2H), where the level of let-7d also varied within the progenitor population. Like in the cortex, let-7d levels were higher in more basally located progenitors compared to those located more apically (Fig. 2H; yellow line demarcates variable levels of let-7d). The higher level of let-7d did not correspond to the small number of Tuj1+ Retinal Ganglion Cells present at this stage, as the greater levels of let-7d extended beyond the Tuj1 expression domain (Fig. 2I). However, cells containing a higher level of let-7d were, in general, located within the region of EdU+ S-phase cells (Figs 2J and S2B). Interestingly, these fluctuations are very dynamic and change over time, and at later stages of development, the cells with the highest levels of let-7d resided at the apical surface (Figs 2K and S2C). Together, this data suggests that let-7 levels fluctuate as cells undergo divisions in the mouse cortex and retina.

### Let-7 activity oscillates during cell division in a neuroblast cell line

**Figure 3:**
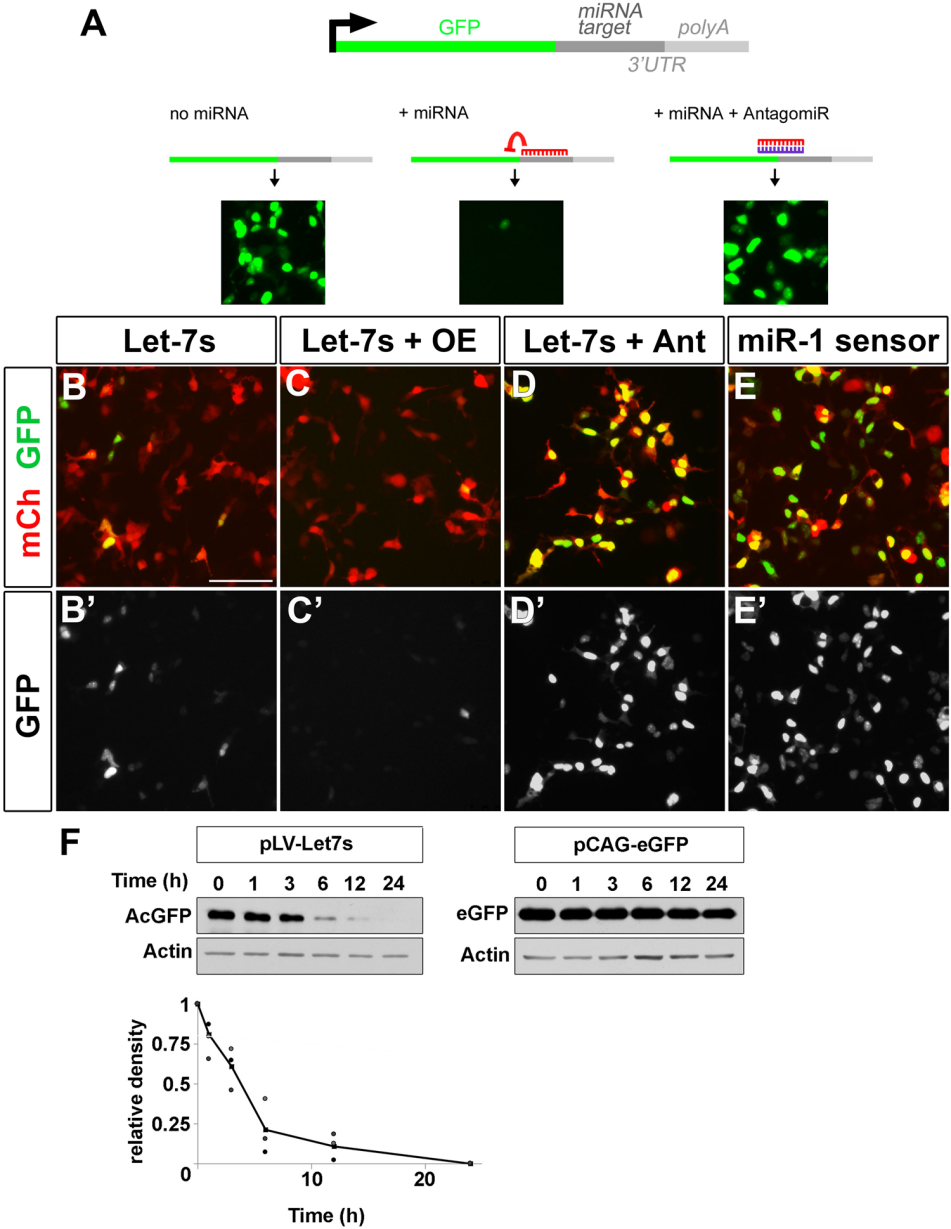
Let-7 is active in human embryonic retina-derived HER10 cells. (**A**) Schematic of a miRNA sensor. miRNA target sites are added to the 3’UTR end of GFP. If the miRNA of interest is present, GFP will be repressed (middle panel in **A**). In contrast, when no miRNA is present (left panel) or when a miRNA antagomiR is added (right panel), GFP is abundantly expressed. **(B**) GFP is present in 20–30−1.64 of HER10 cells transfected with pCAG-mCherry (red) and pLVX-let-7 sensor (let-7s). (**C**) Sensor-GFP expression is repressed when let-7 levels are increased via transfection of pLV-let-7 (OE; compare B’ to C’). (**D)** Co-transfection with a let-7 antagomiR (Ant) leads to GFP expression in almost all transfected cells. **(E)** Nearly all cells transfected with a miR-1 sensor (miR-1s) express GFP (E’). **(F)** Western blot analysis of a cyclohexamide time-course (time (h) = time after cyclohexamide addition) to determine the half-life of AcGFP1 present in the let-7s construct. The half-life of AcGFP1 is short, approximately 3.5 h (left and bottom panels), as compared to eGFP which was > 24 h (right panel). Unaltered images of Western Blots are included in the Supplementary Information (See Fig. S4).

To assay for changes in let-7 activity we used a miRNA sensor. As previously shown^29^, our miRNA sensors are DNA constructs containing acGFP1 (*Aequorea coerulescens* GFP) with two miRNA target sequences at the 3’ end (Fig. 3A). As such, when the miRNA of interest is absent, GFP is abundantly expressed. Conversely, when the miRNA of interest is present and active, it will bind to the target sequences and repress GFP expression. Thus, high GFP expression reflects a lower activity of the miRNA of interest, while low levels of GFP indicate a higher miRNA activity. By treating transfected cells with Cycloheximide to inhibit translation, we confirmed that the half-life of the GFP in our sensor construct was 3.478 h (+/−1.64 h; Fig. 3F), indicating that the stability of the GFP in our assay is low enough to observe rapid and dynamic changes in let-7 activity. We transfected a let-7 miRNA sensor (let-7s) and pCAG-mCherry (as a transfection control) into an immortalized human retinoblast cell line (Ad12 HER10 cells, hereafter referred to as HER10 cells^55,56^). In HER10 cells transfected with let-7s alone, approximately 25% of mCherry+ cells expressed GFP (Fig. 3B). When let-7s was co-transfected with a let-7 overexpression construct (pLV-let-7), GFP expression was repressed (Fig. 3C), while nearly all HER10 cells co-transfected with a let-7d antagomiR expressed a high level of GFP (Fig. 3D). As a control, we also transfected HER10 cells with a miR-1 sensor (miR-1s) and found that nearly all mCherry+ cells were also GFP+, suggesting that HER10 cells have a very low level of endogenous miR-1 activity. It is important to note that our let-7 sensor is not specific to one let-7 isoform (see Fig. S3), and instead reflects the net activity of let-7 family members overall. Together these findings suggest that let-7 is endogenously active in HER10 cells, support the use of our miRNA sensor for assaying let-7 activity, and show that we can effectively overexpress and knock-down let-7 in HER10 cells.

**Figure 4:**
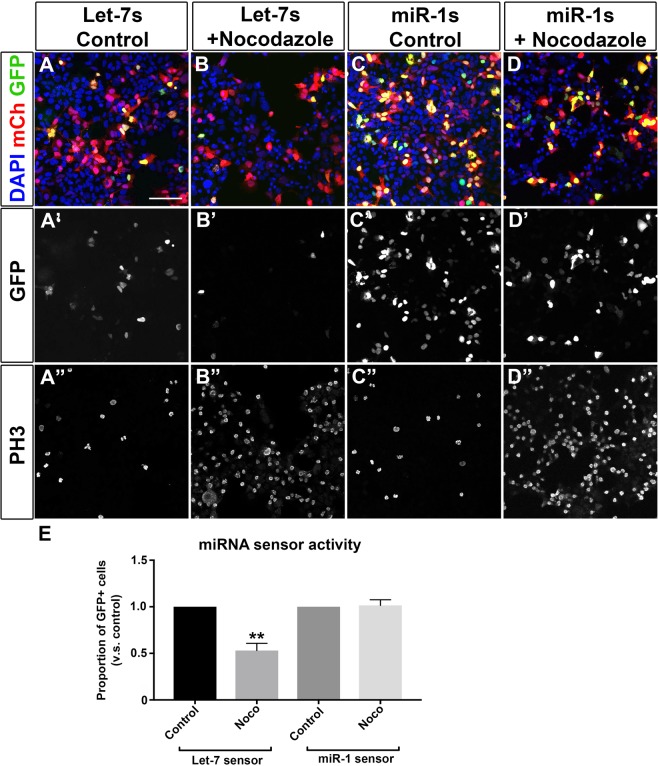
Let-7 activity is high during mitosis in a human embryonic retinoblast line. miRNA activity assessed using miRNA sensors in HER10 cells treated with the microtubule polymerization inhibitor Nocodazole. **(A**,**B)** HER10 cells transfected with let-7 sensor. Approximately 25% of cells were GFP+ in untreated samples (**A**,**A’**), but the number of GFP+ cells decreased when cells were arrested in mitosis by treatment with Nocodazole (B,B’; p = 0.0034, n = 3). An increase in PH3+ cells confirms mitotic arrest in Nocodazole treated samples (compare A” and B”). (**C**,**D**) HER10 cells transfected with miR-1 sensor. The number of GFP + HER10 cells did not change when miR-1 sensor transfected cells were treated with Nocodazole (compare C’ and D’), despite a significant increase in PH3+ cells in Nocodazole- treated samples (compare C” to D”). (**E**) Quantification of the proportion of GFP+ cells in (**A–D**), relative to untreated control samples. n = 3. Scale bar in (**A**) = 50 μm.

To determine whether let-7 activity oscillates during the cell cycle, we co-transfected HER10 cells with either let-7s or miR-1s and pCAG-mCherry and arrested cells in G2/M by overnight treatment with the microtubule polymerization inhibitor Nocodazole^57,58^. Comparing the number of GFP+ mCherry+ cells in Nocodazole-treated cells with controls allowed us to assess changes in the level of let-7 activity when a larger proportion of cells were in mitosis. In untreated samples transfected with let-7s, 23.5% of the cells were GFP+ (Fig. 4A,A’), consistent with our earlier findings (Fig. 3). However, in Nocodazole-treated samples, the percentage of GFP+ transfected cells was significantly decreased (13.3%; Fig. 4B,B’,E). Immunostaining for PH3 after treatment with Nocodazole showed a drastic increase in PH3+ cells compared to controls (compare Fig. 4A”,B”), suggesting that our cell cycle arrest was effective. Treating miR-1s-transfected cells with Nocodazole did not affect the percentage of GFP+ cells (Fig. 4C–E), indicating that this is not the result of a general increase in miRNA activity or an artifact due to Nocodazole treatment.

**Figure 5:**
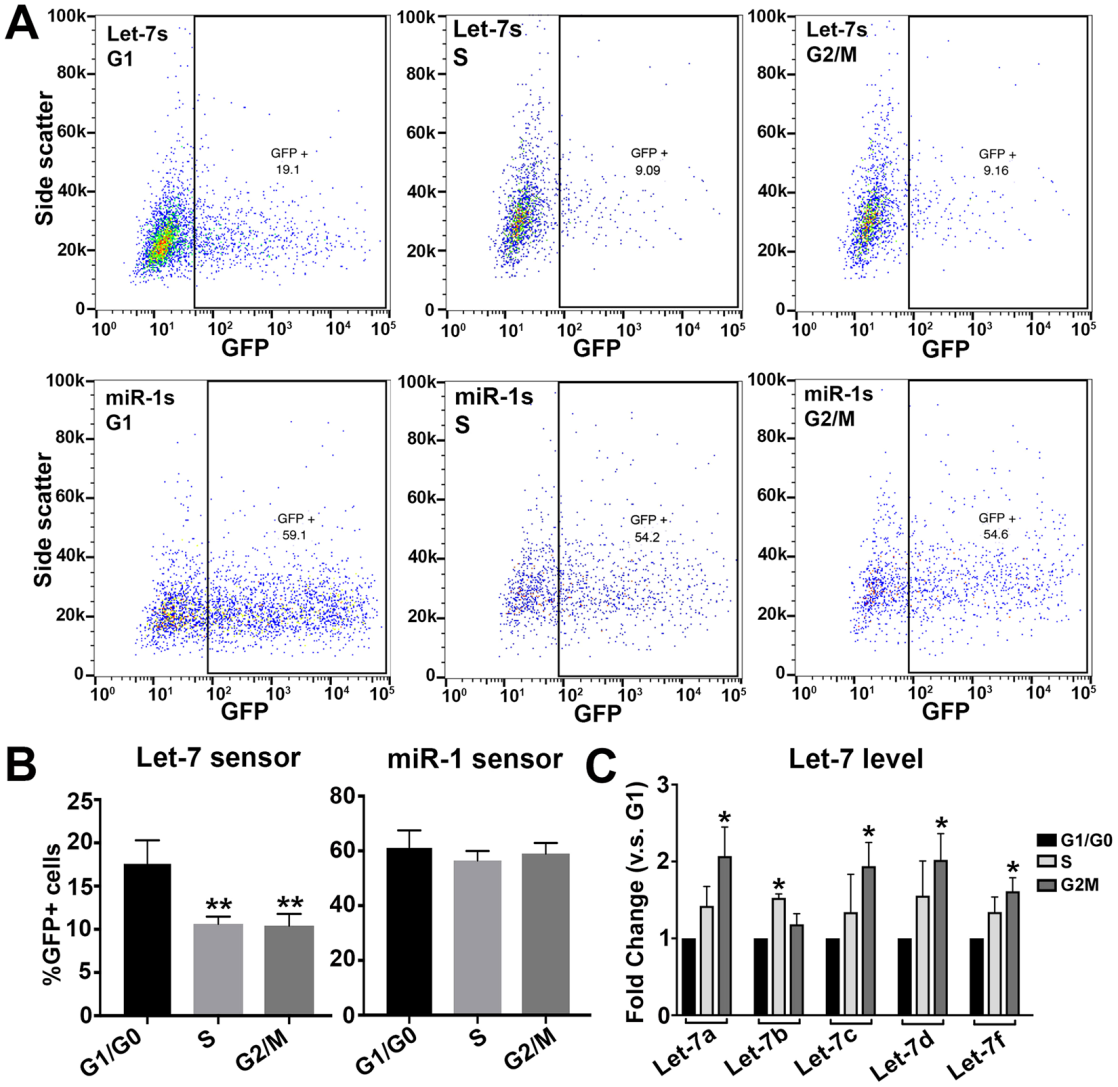
Let-7 activity and levels are highest when cells are in G2/M. (**A**,**B**) miRNA activity in untreated HER10 cells segregated by cell cycle phase using flow cytometry. **(A)** Representative examples of flow cytometry data quantifying the number of GFP+ cells in let-7s-transfected (top panels) or miR-1s-transfected (bottom panels) HER10 cells. The percentage of GFP+ cells in let-7s transfected samples is higher in G1 (black box, upper left panel) as compared to S and G2/M (black boxes, upper middle and right panels). However, the percentage of GFP+ cells in miR-1s transfected samples is similar in all phases of the cell cycle (bottom panels). **(B)** Quantification of flow cytometry data shown in (**A**). N = 3 experiments; 40,000 events per experiment. p = 0.0063 and p = 0.0055 for Let-7s S and G2M (v.s. G1) respectively. (**C**) qRT-PCR analysis of let-7a,b,c,d,f in G1, S, and G2/M phase populations segregated using FACS with ClickIt-EdU and DAPI. With the exception of Let-7b, which is highest in S-phase, all let-7 family members are highest in the G2/M population. N = 3 experiments; with > 500k cells per population. p = 0.012 (let-7a G2M), p = 0.023 (let-7b S), p = 0.039 (let-7c G2M), p = 0.019 (let-7d G2M), and p = 0.039 (let-7f G2M).

To characterize let-7 dynamics during normal cell cycle, without the use of cell cycle inhibitors, we used flow cytometry. First, we transfected HER10 cells with either let-7s or miR-1s (with mCherry as a transfection control) and analyzed GFP expression at different phases of the cell cycle using flow cytometry. Live, sensor-transfected HER10 cells were separated into cell cycle phases (G1, S or G2/M) based on incorporation of the DNA stain Vybrant DyeCycle Violet (DCV). We analyzed 40,000 events per experiment (n = 3 experiments per miRNA sensor), and, on average, our gating strategy considered approximately 6,800 of these events as mCherry+, transfected cells to be used for downstream cell cycle analysis. We were successfully able to resolve transfected samples into the three stages of the cell cycle using DCV (Fig. S5A–C) and analyze the proportion of transfected GFP + cells in each phase (Fig. 5A). The percentage of transfected cells that were also positive for let-7s GFP was lowest in the S and G2/M populations. On average, 17.6% of cells in G1 were GFP+. In contrast, only 10.6% of cells in S, and 10.4% of cells in G2/M were GFP+ (Fig. 5B). There was no significant difference in the percentage of GFP+ cells when comparing the different cell cycle phases in miR-1s transfected HER10 cells (Fig. 5B). Moreover, we used qRT-PCR to quantify the level of individual let-7 isoforms (let-7a, 7b, 7c, 7d, and 7f) in HER10 cells that had been sorted into G1/G0, S, and G2/M cell populations using FACS in combination with ClickIt-EdU and DAPI staining (for gating strategy example, see Fig. S5D). We found that the level of the majority of let-7 isoforms investigated, the exception being let-7b, was significantly increased in the G2/M-phase cell population (Fig. 5C). Taken together, these results suggest that the level and activity of let-7 increases prior to mitosis in HER10 cells.

**Figure 6:**
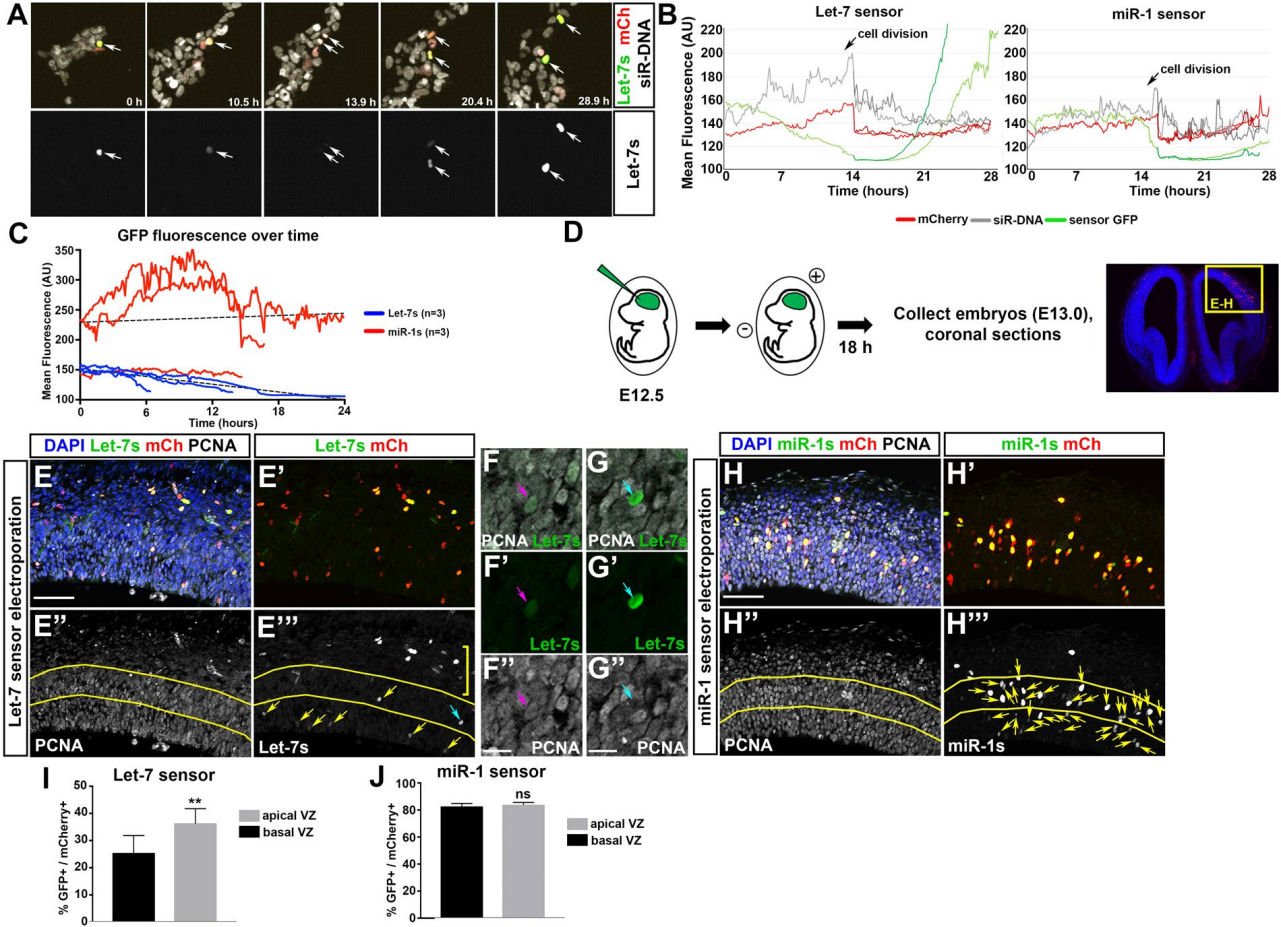
Let-7 activity oscillates when cells progress through the cell cycle *in vitro* and *in vivo*. (**A**–**C**) Real-time assessment of let-7 activity using live imaging. (**A**) Time lapse imaging of HER10 cells transfected with let-7 sensor (green, top panel; white, bottom panel) and mCherry (red). DNA visualized using siR-DNA stain (white, top panel). Following one let-7 sensor GFP+ cell for 28 hours showed that GFP fluorescence is initially high, declines gradually prior to cell division, and increases again in the resulting daughter cells. (**B**) Quantification of let-7 sensor GFP, mCherry and siR-DNA fluorescence over time in the cell shown in A (left panel), with side-by-side comparison of a miR-1 sensor transfected cell (right panel). Unlike let-7 sensor transfected cells, GFP levels remain steady in miR-1 sensor transfected cells until chromosome condensation (which can be visualized by a rapid increase in siR-DNA fluorescence; arrows on graph), during which there is a rapid loss of GFP fluorescence. (**C**) Quantification of GFP fluorescence in three dividing cells in both let-7s (blue) and miR-1s (red) transfected cultures, prior to cell division. **(D**–**J**) Analysis of let-7 activity in the cortex *in vivo* using *in utero* electroporation. (**D**) Schematic describing *in utero* electroporation work-flow. (**E**) Coronal sections of *in utero* electroporated cortex immunostained for GFP (let-7s; green in **E**,E’ and white in E”’), mCherry (red in **E**,E’) and PCNA (white in **E**,E”). The VZ was located by staining for PCNA (yellow lines in E” and E”’). Many GFP+ cells were located in the basal region of the cortex, outside of the VZ (yellow bracket in E”’). There were more GFP+ cells (yellow arrows) in the apical half of the VZ (below the lower yellow line) when let-7s was electroporated. GFP+/PCNA- cells (blue arrow in E”’) were not counted. **(F)** Representative example of a let-7s GFP+ cell (green in F,F’) that is PCNA+ (white in F,F”). (**G**) Representative example of a let-7s GFP+ cell (green in G,G”) that is PCNA- (white in **G**,G”). (**H**) Coronal sections of *in utero* electroporated cortex immunostained for GFP (miR-1s; green in H,H’ and white in H”’), mCherry (red in H,H’) and PCNA (white in H,H”). The VZ was located by staining for PCNA (yellow lines in E” and E”’). There was no difference in the number of GFP+ cells (yellow arrows) located in the apical v.s. basal halves of the VZ when miR-1s was electroporated. **(I)** Quantification of the percentage of electroporated cells (mCherry+) that were GFP+ in the ventral or basal half of the VZ when let-7s was electroporated. N = 3 embryos, p = 0.0071. (**J**) Quantification of the percentage of electroporated cells (mCherry+) that were GFP+ in the ventral or basal half of the VZ when miR-1s was electroporated. N = 3 embryos. Scale bar in (**E**,**H**): 50 μm, scale bar in F and G: 10 μm.

Our flow cytometry analyses of let-7s allowed us to quantitatively analyze let-7 activity within a very large population of cells; however, this still only provided a brief snapshot of a dynamic process. Next, we aimed to follow HER10 cells in real-time to confirm that let-7 activity oscillates in individual cells as they progress through the cell cycle. We transfected HER10 cells with either let-7s or miR-1s and nuclear (H2B-)mCherry and followed them live over 24–36 hours. Using this method, we confirmed that GFP fluorescence from the let-7s oscillates as HER10 cells divide; GFP is high initially but gradually and significantly decreases prior to cell division. GFP later returned in the two resulting daughter cells (Fig. 6A,B and Movie 1). This suggests that let-7 activity is gradually increasing in HER10 cells before they divide, presumably when the cells are in late G2. In contrast, GFP did not fluctuate in miR-1s transfected HER10 cells, and in this case GFP fluorescence was relatively steady, until cell division when miR-1s GFP rapidly declined as the GFP molecules are divided between the two daughter cells (Fig. 6B and Movie 2). We quantified the decrease in GFP fluorescence prior to cell division, in both let-7s and miR-1s transfected cells, over multiple experiments (n = 3) and found that the mean slope of the lines representing GFP fluorescence differed in the let-7s and miR-1s transfected cells (Fig. 6C; p < 0.0001). Together, our let-7s assays suggest that let-7 activity oscillates as retinal progenitor cells progress through the cell cycle.

### Let-7 activity is lower in the apical ventricular zone in the embryonic cortex

Although our live imaging experiments in HER10 cells suggest that let-7 levels oscillate as cells divide *in vitro*, determining whether let-7 levels oscillate in live progenitors within the developing CNS is challenging because endogenous let-7 levels are too high to observe GFP when using our miRNA sensor construct^29^ (and see Fig. S6). To circumvent this complication, we cloned our miRNA sensors into a construct driven by a strong promoter (pCAG-Let-7s and pCAG-miR-1s) and delivered them into the embryonic mouse cortex at E12.5 by *in utero* electroporation (Fig. 6D). After electroporation, we were able to detect GFP from both sensor constructs when the tissue was fixed and immunostained using an anti-GFP antibody. Although we could detect mCherry (our electroporation control), we could not detect GFP in live tissue; thus, we could not perform *ex vivo* live imaging with electroporated samples. However, we did analyze sensor activity in fixed samples 18–24 hours after electroporation (Fig. 6E–H). After electroporation with let-7s, we observed that many of the electroporated cells (mCherry+) that were also GFP + were in the basal region of the developing cortex, where post-mitotic neurons are located (yellow bracket in Fig. 6E”’). We identified VZ progenitors by immunostaining for the progenitor marker PCNA (Fig. 6E”,H”) and found that, in the VZ, few electroporated cells were GFP+ (between 25–35%; Fig. 6I) and many GFP+ cells had relatively low levels of GFP (yellow arrows in Fig. 6E”’). Furthermore, we split the VZ into apical and basal halves (yellow lines in E”,E”’) and found that more GFP+ cells were located in the apical half of the VZ (Fig. 6E”’,I). In contrast, the majority of electroporated cells were GFP+ in miR-1s-electroporated samples (80%; Fig. 6H,J), and there was no difference in the percentage of GFP+ cells when comparing apical/basal regions of the VZ. The results of our let-7s *in utero* electroporations suggest that let-7 activity within the VZ is lower in apically-located cortical progenitors. This finding supports both our *in situ* hybridization profile showing low levels of let-7 in apically-located cells at E13.5, and our hypothesis that let-7 activity oscillates as cells divide.

### Manipulating let-7 alters cell cycle dynamics in HER10 cells

**Figure 7:**
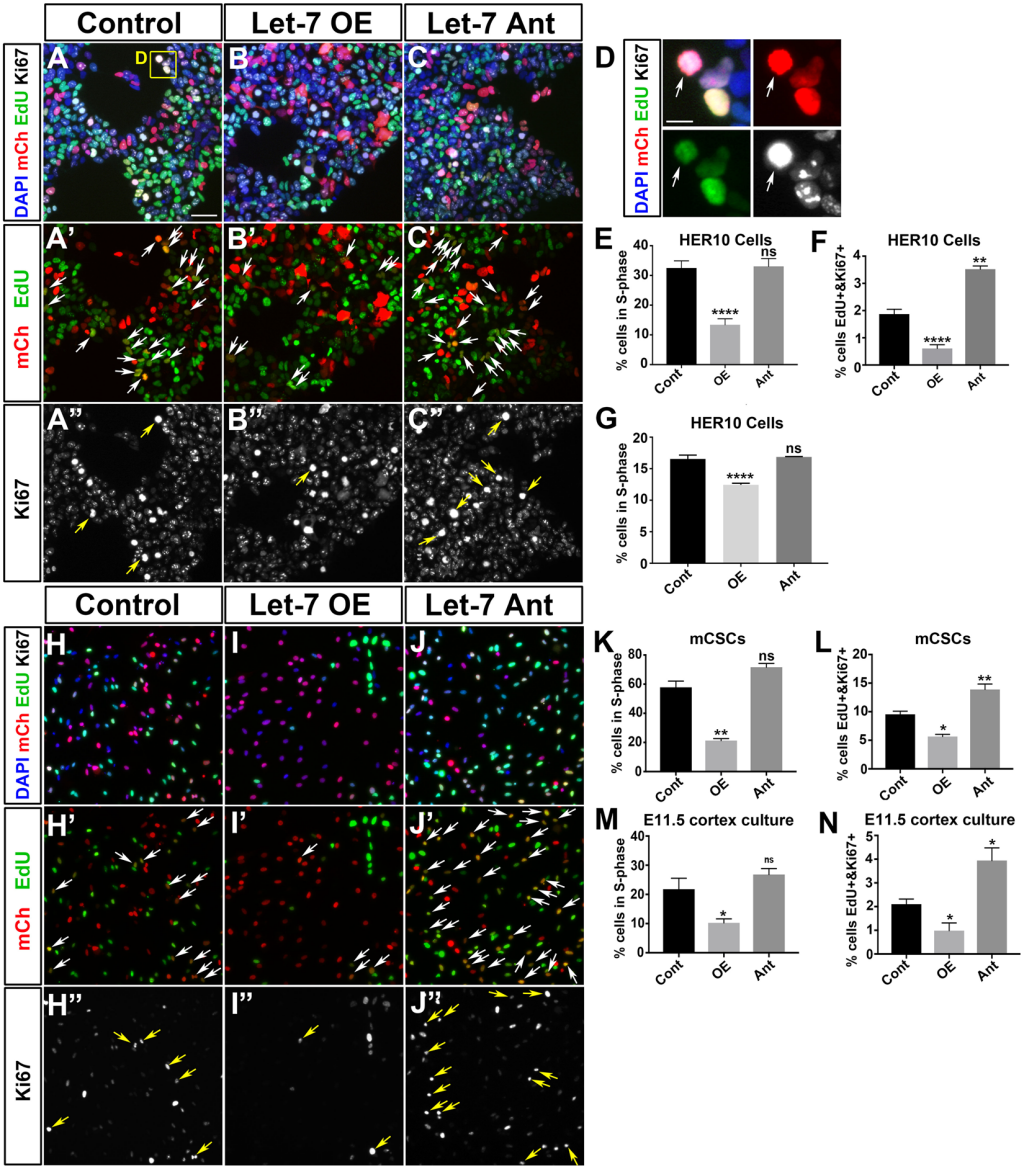
Manipulation of let-7 levels alters cell cycle dynamics in HER10 cells and mCSCs. (**A**–**C**) HER10 cells transfected with mCherry alone (Cont; **A**-A”), pLV-let-7 (let-7 OE; B-B”) or a let-7 antagomiR (let-7 Ant; C-C’). mCherry+ cells are shown in red (A-C and A’-C’), EdU+ in green (**A–C** and A’-C’), and Ki67+ in white (A–C and A”-C”). DAPI is shown in blue (**A**–**C**). White arrows in A’-C’ indicate transfected EdU+ cells. Yellow arrows in A”-C” indicate transfected cells that are EdU+ and Ki67+. **(D)** Close up of the yellow box shown in (A). Example of mCherry+, EdU+, and Ki67+ cell (white arrow). **(E)** Quantification of mCherry+ and EdU+ HER10 cells shown in A-C. n = 4, p = 0.0001 for let-7 OE. **(F)** Quantification of EdU+ and Ki67+ cells shown in (A-C). n = 4, p = 0.0001 and p = 0.0019 for let-7 OE and Ant, respectively. **(G)** Quantification of flow cytometry analysis in HER10 cells transfected with let-7 OE or Ant and stained with ClickIt EdU and DAPI. The number of cells in S-phase is reduced in OE-transfected samples. N = 3, 40,000 cells per experiment; p = 0.0004 **(H**–**L)** mCSCs transfected with mCherry (Cont; **H**-H”), let-7d mimic (let-7 OE; **I**-I”) or let-7 antagomiR (let-7 Ant; **J**-J’). mCherry+ cells are shown in red (**H**–**J** and H’-J’), EdU+ in green (**H**–**J** and H’-J’), and Ki67+ is shown in white (**H**–**J** and H”-J”). DAPI in blue (**H**–**J**). White arrows in H’-J’ indicate EdU+, transfected cells. Yellow arrows in H”-J” indicate transfected cells that are both EdU+ and Ki67+. **(K)** Quantification of mCherry+ and EdU+ mCSCs shown in H-J. n = 3, p = 0.0013 for let-7 OE. **(L)** Quantification of EdU+ and Ki67+ cells in (H-J). n = 3, p = 0.0112 and p = 0.0067 for let-7 OE and Ant, respectively. **(M)** Quantification of mCherry+ and EdU+ in similar experiments performed in E11.5 mouse cortex primary cultures. n = 3, p = 0.0374 for let-7 OE. **(N)** Quantification of mCherry+ and EdU+ in similar experiments performed in E11.5 mouse cortex primary cultures. n = 3, p = 0.0405 and p = 0.0231 for let-7 OE and Ant, respectively. Scale bar in (**A**) = 50 μm. Scale bar in (**D**) = 20 μm.

Given that let-7 oscillates as cells progress through the cell cycle, we next wanted to determine whether manipulating the level of let-7 conversely alters cell cycle dynamics. We performed sequential EdU incorporation and Ki67 immunostaining in HER10 cells which either overexpressed let-7 (by transfection with pLV-let-7; let-7 OE) or in which let-7 was knocked down using the let-7 antagomiR (let-7 Ant). 24 hours after manipulation of let-7 levels, HER10 cells were given a 30-min pulse of EdU to label cells in S-phase. EdU was then washed out, and after a subsequent 18-hour incubation, the cells were fixed, EdU was detected, and the samples were stained for Ki67 to label actively dividing cells. If a mCherry+, transfected cell was EdU+, this suggests that this cell was in S-phase 24 hours after let-7 manipulation, while EdU+ cells that were also Ki67+ would indicate the population of cells that were in S-phase 24 hours after manipulation and were in mitosis 18 hours later. We used this number to provide a read-out of cell cycle progression. In HER10 cells, the percentage of cells in S-phase 24 hours after transfection was similar in control- and let-7 Ant-transfected samples (32.5% in control- vs. 33.0% in let-7 Ant-transfected samples were mCherry + EdU+; compare Fig. 7A’,C’,E). However, the number of EdU+ cells in let-7 OE-transfected HER10 cells was significantly reduced compared to control (13.4% in let-7 OE- transfected cells; compare Fig. 7A’,B’,E). Likewise, the percentage of EdU+ Ki67+ cells was reduced in let-7 OE-transfected cells compared to controls (1.87% in control v.s. 0.61%; compare Fig. 7A”,B”,F). In contrast, the percentage of EdU + Ki67+ cells was significantly increased in let-7 ant-transfected HER10 cells (3.52% in let-7 Ant-transfected cells; compare Fig. 7A”,C”,F).

In addition to immunolabeling analyses, we used flow cytometry to quantify the number of HER10 cells in each phase of the cell cycle in let-7 OE- or let-7 Ant-transfected samples. Transfected HER10 cells were given a 30-min pulse of EdU just prior to collection, fixed, and stained using the ClickIt-EdU detection kit. Cells were analyzed by flow cytometry for ClickIt-EdU and DAPI (for gating strategy, see Fig. S5D). We found that there was a reduction in the S-phase population when comparing control-transfected cells to cells overexpressing let-7 (25.2% of cells in S-phase in control, vs. 20.2% in let-7 OE, p = 0.0004; Fig. 7G). We did not observe a difference in the number of cells in S-phase by flow cytometry when let-7 was knocked down using let-7 Ant (Fig. 7G).

### Manipulating let-7 alters cell cycle dynamics in cortical progenitors

Next, we wanted to determine whether these findings were consistent in a model more similar to endogenous CNS progenitors. To this end, we first obtained commercially available mouse cortical stem cells isolated from E14.5 mouse brain (Roche; hereafter referred to as mCSCs) and, secondly, we generated primary cultures from mouse cerebral cortex at E11.5. Transfection of our primary cultures (Fig. S6A–D) or mCSCs (not shown) with the let-7 miRNA sensor resulted in a very small number of cortical progenitors that were positive for both the progenitor marker Sox2 and our sensor GFP. This suggests that cortical progenitors in our culture systems have a high enough level of endogenous let-7 activity to detect GFP from the sensor construct. Furthermore, many of the GFP+ cells in our primary cultures were Tuj1+ (Fig. S6E,F), suggesting that some of the GFP expression observed in our primary cultures may be due to a subset of post-mitotic neurons that reduce let-7 activity upon differentiation. Because let-7 sensor GFP was highly repressed in primary cultures, we could not repeat the let-7 sensor activity assays we performed in HER10 cells. The high level of endogenous let-7 activity is consistent with our *in situ* hybridization findings, which suggest that by E13.5, let-7 levels are already high in cortical progenitors (Fig. 1).

Despite the high level of let-7 in cortical progenitors, co-transfection of the sensor with let-7 Ant increased GFP expression (Fig. S6G,H), suggesting that we can effectively reduce let-7 activity in our culture models and use these systems to investigate how let-7 regulates cell cycle dynamics. Thus, we performed sequential EdU incorporation and Ki67 immunostaining (as described above) in mCSCs and E11.5 cortex primary cultures transfected with either a let-7 mimic (let-7 OE) or let-7 Ant (Fig. 7H–N). Similar to HER10 cells, when let-7 was knocked down in either mCSCs or E11.5 mouse cortical progenitors, there was no significant difference in the number of EdU+ cells in S-phase 24 hours after transfection (Fig. 7K,M). Additionally, similar to HER10 cells, let-7 overexpression significantly reduced the number of cells in S-phase in both culture systems (Fig. 7K,M). Furthermore, the number of cells that were EdU + Ki67+ was significantly decreased in both culture systems upon transfection with let-7 mimic (Fig. 7L,N) and increased in both culture systems with let-7 Ant (Fig. 7L,N). This confirms that, similar to HER10 cells, increasing let-7 reduces the number of cells in S-phase and decreases cell cycle re-entry while reducing let-7 activity promotes cell cycle re-entry.

### Let-7 mediates cell cycle kinetics by controlling cell cycle length

**Figure 8:**
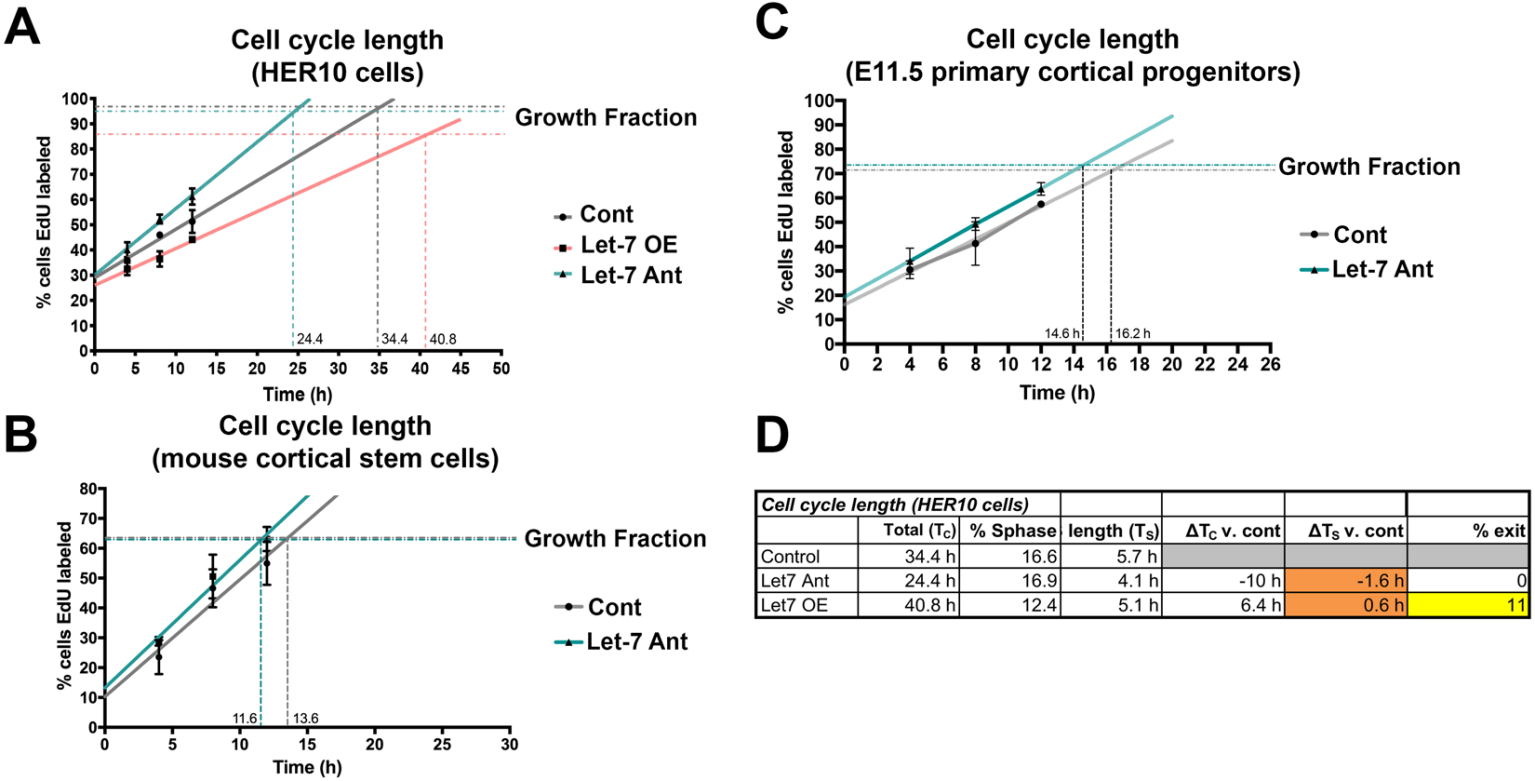
Let-7 regulates cell cycle dynamics by altering the length of the progenitor cell cycle. Cumulative EdU labeling in HER10 cells (**A**), mCSCs (**B**) and E11.5 mouse cortex primary cultures (**C**), to assess the length of the progenitor cell cycle. (**A**) HER10 cells were transfected with mCherry alone (Cont; grey line), pLV-let-7 (Let-7 OE; orange line) or a let-7 antagomiR (Let-7 Ant; teal line). The percentage of EdU+ cells was visually quantified at 4-, 8-, 12-, 24- and 48-hour time points (after addition of EdU). n = 3 samples per condition. Growth fraction = the percent of EdU+ cells at the final time point. (**B**) Cumulative EdU labeling in mCSCs transfected with mCherry alone (Cont; grey line) or a let-7 antagomiR (Let-7 Ant; teal line). The percentage of EdU+ cells was visually quantified at 4-, 8-, 12-, and 24-hour time points (after addition of EdU) n = 3 samples per condition. (**C**) Cumulative EdU labeling in E11.5 mouse cortex primary cultures transfected with mCherry alone (Cont; grey line) or a let-7 antagomiR (Let-7 Ant; teal line). The percentage of EdU+ cells was visually quantified at 4-, 8-, 12-, and 24-hour time points (after addition of EdU. n = 3 samples per condition.) (**D**) Table comparing the total cell cycle length in HER10 cells from each condition (T_C_) and the percent of cells in S-phase from our flow cytometry analysis (see Fig. 7B). We used these observations to estimate the length of S-phase (T_S_). We predict that the length of S-phase changes with Let-7 OE and Let-7 Ant (∆T_S_), but that this does not explain the total changes in cell cycle length (∆T_C_). On average, in let-7 OE transfected cultures, 11% of cells exit the cell cycle.

To gain a more complete understanding of how let-7 is regulating cell cycle dynamics, we performed cumulative EdU labeling, an established method for measuring cell cycle length^5,28^. We transfected HER10 cells with our let-7 OE construct or the let-7 Ant (and H2B-mCherry as a transfection control) and provided a continuous supply of EdU in the culture media. The samples were collected at 4, 8, 12, 26 and 50-hour intervals, fixed, and stained for EdU (Fig. 8A). We calculated that, in control HER10 cells (transfected with mCherry only), the length of the cell cycle was 34.4 h. The length of the cell cycle was drastically reduced when let-7 was knocked down: in this case, the calculated cell cycle length was 24.4 h, a 29% reduction. In contrast, the length of the cell cycle was increased when let-7 was overexpressed, with the calculated cell cycle length being 40.8 h (a 19% increase). Notably, the maximum level of EdU+ cells reached at the end of the experiment (the “growth index”) was approximately 10% lower in the let-7 overexpressing cells (85.7% in let-7 OE compared to 96.7% and 94.4% for control and let-7 Ant, respectively), suggesting that some cells exited the cell cycle when let-7 was overexpressed. Additionally, we measured the effect let-7 knock down had on cell cycle length in mCSCs and E11.5 mouse cortex primary cultures (Fig. 8B,C). We observed a similar decrease in cell cycle length when let-7 was knocked down in cortical progenitors (a 15% and 10% reduction in total cell cycle length in mCSCs and E11.5 cortex primary cultures, respectively). Interestingly, transfecting mCSCs with the let-7 mimic resulted in a dramatic number of cells exiting the cell cycle (less than 30% of cells were EdU+ at the final time point; Fig. S7), making our calculations of cell cycle length complicated in this condition. Together, these results suggest that let-7 regulates cell cycle dynamics, by controlling both cell cycle exit and mediating the length of the neural progenitor cell cycle.

### Let-7 regulates the cell cycle during S/G2

**Figure 9:**
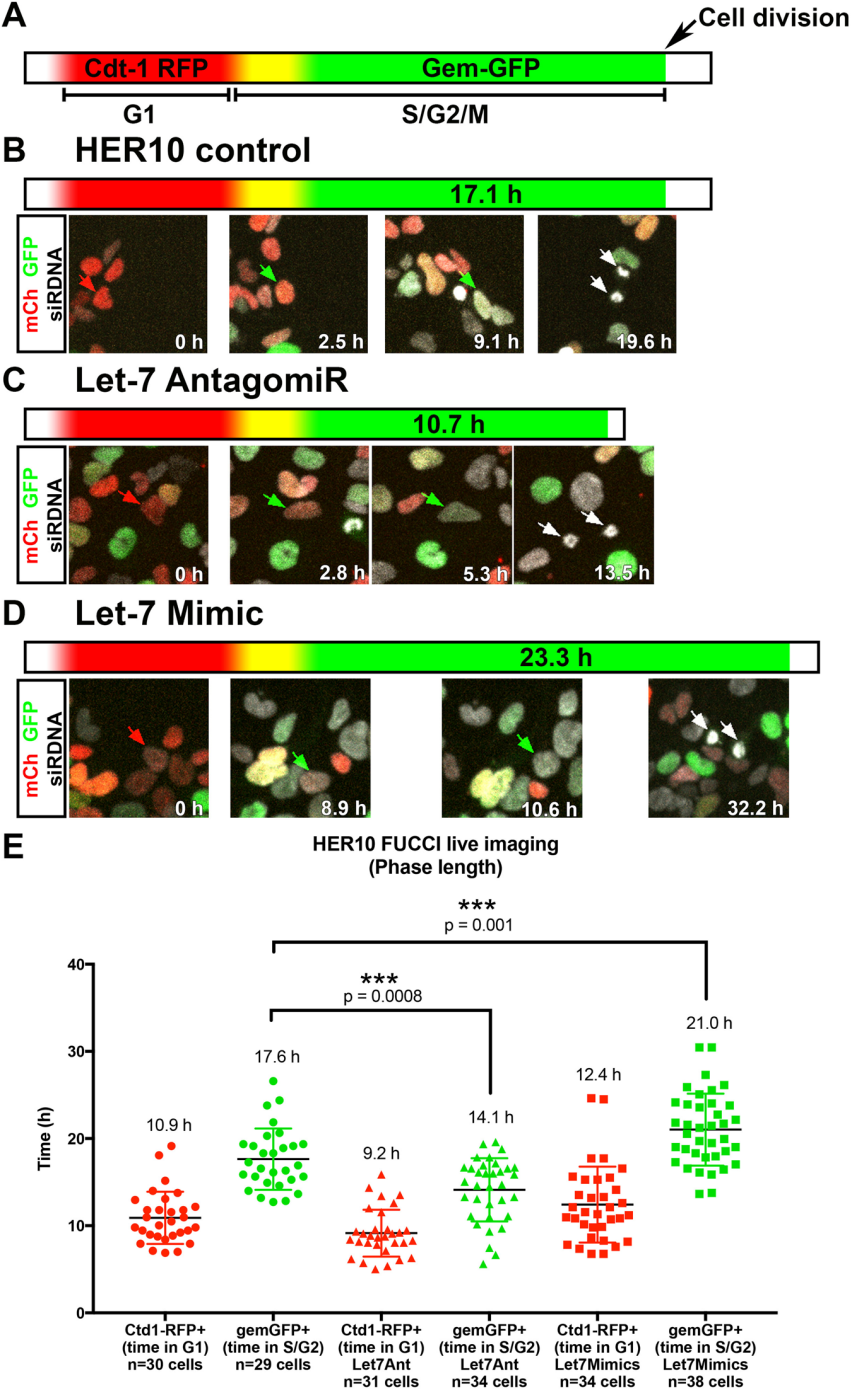
The length of S/G2/M is affected by let-7 overexpression and knockdown. Real-time assessment of cell cycle phase length by live-imaging using the fluorescent reporter FUCCI. **(A)** Schematic of the FUCCI reporter. Cells express Cdt-1 RFP (red) in G1. Upon entry into S-phase, cells begin to express Gem-GFP and are yellow for a short period before Cdt-1 RFP is degraded and the cells are green. **(B**) Representative example of a movie assessing the length of S/G2/M in control HER10-FUCCI cells. The cell being tracked (red arrow) is red at 0 h. After 2.5 h, Gem-GFP becomes detectable (green arrows). The cell remains green for 17.1 h before dividing into two daughter cells (white arrows; see Movie 3). **(C)** Representative example of a movie assessing the length of S/G2/M in let-7 Ant-transfected HER10-FUCCI cells. In this example, the Gem-GFP+ phase is only 10.7 h (green arrows) before the cell divides into two daughter cells (white arrows; see Movie 4). **(D)** Representative example of a movie assessing the length of S/G2/M in let-7 mimic transfected HER10-FUCCI cells. In this example, the Gem-GFP+ phase is extended to 23.3 h before the cell divides (white arrows; see Movie 5). **(E)** Quantification of all HER10-FUCCI live imaging. (n = 3 movies per condition, with approximately 30 cells tracked per phase (G1 or S/G2/M), per condition).

To gain insight into the mechanism of how let-7 regulates cell cycle length, we used a fluorescent reporter, Fluorescence Ubiquitination-based Cell Cycle Indicator (FUCCI^59^), to measure the length of each phase of the cell cycle in live HER10 cells. The FUCCI reporter contains Cdt1-RFP (which is expressed during G1) and Geminin-GFP (Gem-GFP), which is turned on as soon as cells enter S-phase (Fig. 9A). Thus, cells are red for the extent of G1, and as soon as GFP is detectable (over 5% of background levels for the green channel) we considered them in S/G2/M until the cell divides. Using this reporter, we determined that the average length of G1 in control HER10-FUCCI cells was 10.9 h and the average length of S/G2/M was 17.6 h (Fig. 9B,E), making the total length of the cell cycle approximately 28.5 h. Taking into consideration the time when FUCCI-cells are colorless before accumulation of Cdt-1 RFP, this is close to our estimation of the cell cycle length by cumulative EdU labeling, which was 34.4 h (Fig. 8A). When let-7 was knocked down using the let-7 Ant, the length of G1 in HER10-FUCCI cells did not significantly change (on average, G1 lasted 9.2 h), while the length of S/G2/M was significantly shorter (14.1 h, p = 0.0008; Fig. 9C,E). Likewise, the length of G1 was not significantly different in let-7 mimic-transfected HER10-FUCCI cells (on average, G1 lasted 12.4 h). However, S/G2/M was significantly longer when let-7 was overexpressed (on average, S/G2/M lasted 21.0 h, p = 0.001; Fig. 9D,E). In these experiments, we could not use a transfection control, and cannot not distinguish between transfected and non-transfected cells; thus, our results likely reflect an underestimation of the actual affect let-7 has on cell cycle length. Nonetheless, the estimated length of the total cell cycle from our let-7 Ant and OE FUCCI experiments (23.3 h and 33.4 h, respectively), is close to those we estimated from cumulative EdU labeling (Fig. 8).

## Discussion

In the developing CNS, neural progenitor cell cycle progression and exit are highly regulated and synchronized with developmental time^1–3^. The basic components of the cell cycle machinery are essential regulators of neural progenitor proliferation, and their roles in regulating cell cycle dynamics are well established. Less is known about the molecules that provide progenitor cells with a sense of temporal identity and coordinate changes in cell cycle dynamics as development progresses. Recent work has emphasized the importance of miRNAs in regulating developmental transitions in a diverse array of model systems, including the developing CNS^21,22^. However, defining their exact role in controlling cell proliferation has been challenging, given that proliferation and differentiation are interdependent, and because much of what we know about the role of miRNAs in the developing CNS involves studies using animals that lack all mature miRNAs, which can lead to confusing results as different miRNAs might have opposing roles and excessive cell death can also hinder some findings.

Here, we focus on the role of one miRNA family, let-7, in regulating the cell cycle kinetics of neural progenitors. Our *in situ* hybridization profile suggests that the level of let-7 is spatially and temporally dynamic throughout the developing CNS. For instance, let-7 is transiently highly expressed in the earliest differentiated neurons of the telencephalon (preplate) but at E13, as the preplate splits into marginal zone and the subplate, these neurons exhibit much lower levels of let-7, suggesting that perhaps let-7 downregulation is important to allow for neural migration and preplate splitting. Importantly, early in development, at E11.5, let-7 levels are low in neural progenitors but gradually increase over time (Fig. 1A–D). This is consistent with an earlier report showing that the level of let-7, in addition to miR-9 and miR-125b, increases in retinal progenitors as development progresses^29^. Our study supports that this progressive increase in let-7 is conserved in neural progenitors in many regions of the CNS. Here, we show that let-7 levels rise dramatically in progenitors in the mouse cortex, retina, hippocampus, and cerebellum; suggesting a conserved developmental program including let-7, and perhaps miR-9 and miR-125b, likely regulate developmental timing in neural progenitors throughout the CNS.

One of the most striking findings from our study is that the level and activity of let-7 oscillate as cells divide. This observation is apparent by *in situ* hybridization in the cortex (at E13.5; Fig. 2A–E,G) and the retina (at E11.5; Fig. 2H–K). At these specific developmental stages, let-7d levels vary within the neural progenitor population and correlate to the location of cell bodies as they progress through IKNM: let-7 is high in regions where EdU+, S-phase cells reside and lower in apically-located PH3+ mitotic cells. This phenomenon is only observable at a brief developmental time, perhaps when the dynamic range of the *in situ* hybridization detection allows it. Our let-7 miRNA sensor experiments further show that the activity of let-7 also oscillates as cells progress through the cell cycle. This is most apparent in our live imaging experiments, which show the activity of let-7 increases as cells prepare to divide (Fig. 6, Movies 1 and 2). The increasing level of let-7 activity prior to cell division in HER10 cells seems to oppose our *in situ* findings, which show mitotic cells having a low level of let-7 at E11.5 in the retina (and E13.5 in the cortex; Fig. 2). One explanation is that HER10 cells, being an immortalized cell line isolated from human embryonic retinoblasts at 15–19 weeks^60^, are more representative of progenitors at a later developmental stage. It is important to note that, by *in situ*, we see a high level of let-7 in apically-located neural progenitors at late developmental stages, which is consistent with our HER10 cell live imaging. It is possible that the level of let-7 also oscillates at later developmental time points, but that these fluctuations may be outside of the detectable range for *in situ* hybridization to resolve. Importantly, our E12.5 let-7 sensor *in utero* electroporations suggest the activity of let-7 is lower in apical progenitors, which is consistent with the fluctuations we see in let-7d *in situs* in the cortex at E13.5 (Fig. 2). Taken together, our data suggest that let-7 activity fluctuates in neural progenitors when cells divide, but we propose that this process is temporally dynamic.

It is intriguing to speculate that miRNAs may be key regulators of ultradian oscillations that drive biological clocks throughout development, because short period oscillations in miRNA levels have also been identified for other miRNAs. For example, an oscillating, negative feedback loop between miR-9 and Hes transcription factors, downstream effectors of the Notch signaling pathway, control the balance between proliferation and differentiation in neural stem cells^61,62^. Oscillation in Notch signaling activity is a well-established mechanism driving developmental timing in a number of different systems, including the segmentation clock that is active during somitogenesis and neural progenitor differentiation^63,64^. Let-7 itself has been shown to oscillate in another context: its expression fluctuates with the circadian cycle in *Drosophila*, and here let-7 also inversely regulates the length of the circadian rhythm^65^. Notably, there is precedence for the cell cycle-dependent regulation of miRNAs. Indeed, CyclinD1 can directly regulate the expression of the miRNA processing enzyme Dicer^66^, suggesting that cell cycle-mediated changes in Dicer activity may impact miRNA oscillation. Investigating transcriptional regulators of let-7 expression and identifying novel regulators of let-7 processing and activity will help shed light on the factors controlling let-7 oscillations.

The oscillatory behavior of let-7 suggests that its expression and/or activity is regulated by the cell cycle; but here, we also provide evidence that let-7 regulates cell cycle kinetics. Experimentally manipulating let-7 levels has profound impacts on the cell cycle: overexpression lengthens the cell cycle and promotes cell cycle exit, while inhibiting let-7 activity using an antagomiR shortens the progenitor cell cycle. This is true in an immortalized retinal cell line and two separate models for cortical progenitors. Given that let-7 levels rise in progenitors of the retina (Fig. 1)^24,29,67^, cortex^41^, cerebellum, and hippocampus (Fig. 1) as development progresses, and that the length of the neural progenitor cell cycle concurrently increases^5,9,10^, we propose that let-7 is one factor regulating progenitor cell cycle lengthening in the developing CNS. Our FUCCI experiments further indicate that let-7 is specifically affecting S/G2 progression while no significant changes are observed in G1. This observation correlates with classic experiments using 3H-thymidine cumulative labeling to calculate the lengthening of cell cycle during normal retinal development^5^, suggesting that changes in cell cycle length during development are in part due to an increase in S-phase length. Taking into consideration our calculations of total cell cycle length from cumulative labeling experiments (T_C_; Fig. 8D), and our flow cytometry-based estimation of the percentage of HER10 cells in S-phase at a given time (Fig. 7G), we can estimate the length of S-phase in each condition (T_S_). We predict that, although S-phase does change relative to controls in both let-7 OE and let-7 Ant (∆T_S_; orange in Fig. 8D), this relatively small change does not account for the total change in cell cycle length (compared to the control condition; ∆T_C_). In combination with our FUCCI experiments, our data suggests that let-7 likely regulates multiple cell cycle phases, including S and G2 phases. Further experiments, like identifying let-7 targets in neural progenitors, could confirm our predictions, and would provide insight into the molecular mechanisms underlying neural progenitor cell cycle lengthening *in vivo*.

## Methods

### Mice

Pregnant CD-1 females were obtained from Charles River and housed until embryos or neonates were at the proper developmental stage for dissection and fixation. All animals were used with approval from the University of California Davis Institutional Animal Care and Use Committees and housed and cared for in accordance with the guidelines provided by the National Institutes of Health. For EdU incorporation in mouse embryos, pregnant CD-1 females were injected with 0.5 mg EdU per 10 g body weight. After 90 minutes of EdU incorporation, dams were sacrificed and embryos were dissected and fixed for ClickIt Detection or *in situ* hybridization.

### HER10 cell line

Ad12HER10 immortalized human retinoblast cells (HER10 cells^55,56^) were a gift from Dr. Tom Glaser. HER10 cells were maintained in Dulbecco’s Modified Eagle’s Medium (DMEM; Gibco) supplemented with 10% heat-inactivated Fetal Bovine Serum (FBS; Life Technologies) and penicillin-streptomycin (P/S; Gibco) and passaged with 0.25% Trypsin-EDTA (Gibco) weekly. We generated a stably-expressing HER10-FUCCI cell line by transfecting HER10 cells with ES-FUCCI (Addgene plasmid # 62451; http://n2t.net/addgene:62451; RRID:Addgene_62451) and culturing in the presence of Hygromycin B (100 μg/mL).

### Mouse cortical stem cells and E11.5 cortex primary culture preparation and transfection

We obtained commercially available mouse cortical stem cells (mCSCs; Roche) and cultured them according to manufacturer’s recommendations. E11.5 cortex was dissected from CD-1 embryos and cells were dissociated with Accutase for 1 min at RT. Dissociated cells were plated on glass coverslips coated with basement membrane extract (BME, 1:30 dilution; Trevigen), at a density of approximately 2 brains per 3 coverslips, in cortical stem cell media (DMEM/F12 supplemented with glucose, L-glutamine, NaHCO3, N-2 supplement, 0.02 ng/mL FGF/EGF and P/S). mCSCs and primary cultures were transfected with RNA and either 5 nM let-7 mimic (Dharmacon) or 5 nM let-7 antagomiR (Exiqon) using Lipofectamine 2000 (Invitrogen) according to manufacturer’s protocol. RNA used for transfection (mCherry or miRNA sensors) was transcribed using mMessage mMachine T7 ULTRA (Ambion) according to manufacturer’s protocol.

### miRNA *in situ* hybridization and immunohistochemistry

Embryos and neonates were collected from pregnant CD-1 females at different stages, ranging from E11.5-P0 (birth) and whole heads, eyes, or brains were fixed in a modified Carnoy’s fixative overnight at 4 °C. After fixation, samples were dehydrated into 100% ethanol (stepwise) overnight at 4 °C and embedded in paraffin. Horizontal sections of whole embryo heads (E11.5-E16.5), sagittal sections of whole eyes (P0), or coronal sections of whole brains (E18.5, P0) were prepared at 7 μm, collected onto SuperFrost slides and air dried overnight at room temperature. A double digoxigenin (DIG)-labeled, locked nucleic acid (LNA) *in situ* hybridization probe (miRCURY LNA Detection probe; hsa-let-7d) was purchased from Exiqon (Woborn, MA). *In situ* hybridization was performed using the miRCURY LNA microRNA Detection FFPE microRNA ISH Optimization Kit 4 (Exiqon), which includes hybridization buffers and control probes (LNA scramble microRNA probe and LNA U6 snRNA control probe), according to manufacturer’s protocol. LNA probes at the following concentrations: let-7 (c and d; 40 nM), scramble (40 nM), U6 (5 nM), and miR-183 (40 nM) were hybridized for 1 hour at 55 °C. Detection was performed using an alkaline phosphatase conjugated anti-DIG secondary antibody (Roche) and NBT (nitroblue tetrazolium)/BCIP (5-bromo-4-chloro-3-indolyl phosphate) stock solution (Roche). After the reaction was deemed complete, slides were fixed with 4% paraformaldehyde and either used for immunostaining or directly coverslipped with Fluoromount-G (Southern Biotech, Birmingham, AL). Slides were washed with PBS-0.1% TritonX-100, blocked in PBS-0.1% TritonX-100 supplemented with 10% Donkey Serum (Jackson Laboratories) and immunostaining was performed via overnight incubations at 4 °C using the following antibodies diluted in blocking solution: rabbit anti-Pax6 (1:100; Biolegend), goat anti-Lhx2 (1:200; Santa Cruz), rabbit anti-Lin28 (1:200, Abcam), rabbit anti-Phosphohistone H3 (PH3, 1:100; Thermo Fisher Scientific), and mouse anti-βIII tubulin (1:500; Biolegend). Primary antibodies were visualized using the appropriate Alexa Fluor-conjugated secondary antibodies (1:200; Thermo Fisher Scientific). When applicable, EdU was detected using the ClickIT EdU Alexa Fluor 647 detection kit (Invitrogen). Bright field and fluorescent images were taken on a Nikon Eclipse E800 microscope using Olympus cellSens Dimensions software. Images were assembled in Adobe Photoshop.

### Protein stability assays

HER10 cells were plated at approximately 6 × 10^5^ cells in 35-mm dishes and incubated in a CO2 incubator overnight. Next, cells were transfected with the let-7 miRNA sensor or pCAG-GFP using Lipofectamine 2000 (Invitrogen) according to manufacturer’s protocols. 24 hours later, the media was replaced with complete media supplemented with 20 μg/ml of Cycloheximide. Cell lysates were collected at t = 0, 1, 3, 6, 12, and 24 later in lysis buffer [50 mM HEPES (pH 7.5), 150 mM NaCl, 1.5 mM EGTA, 10% glycerol, 1% Triton X-100, 2 mM PMSF, 10 μg/ml leupeptin, 1 μg/ml aprotinin, 10 mM NaF and 1 mM orthovanadate] and supernatants were obtained by centrifugation at 13,000 rpm, at 4 °C for 15 min. Western blotting was carried out using a rabbit anti-GFP antibody diluted 1:1000 (Invitrogen) and appropriate secondary antibodies following standard protocols described elsewhere^68,69^.

### *In vitro* let-7 sensor assays

To assay for let-7 activity (and miR-1 activity as a control) we used miRNA sensors (pLVx-let-7s or pLVX-miR-1s^29^), which were a gift from Dr. Tom Reh (University of Washington). HER10 cells were transfected in a 24-well plate with 0.5 μg miRNA sensor constructs and 0.5 μg pCAG-mCherry using Lipofectamine 2000 (Invitrogen) according to manufacturer’s protocols. After transfection, transfection media was replaced with maintenance media supplemented with 40 μM Nocodazole (Sigma-Aldrich) and incubated for 18 hours. After Nocodazole treatment, cells were immediately fixed in 4% paraformaldehyde for 10 minutes at RT and washed with PBS for subsequent analysis. The efficiency of Nocodazole treatment was confirmed by immunostaining for PH3, as described above. Fixed and stained cells were imaged on a Leica DM 5000 M fluorescence microscope with a Leica DFC 500 camera using the same exposure times to compare control and Nocodazole treated samples. Quantitation was performed by counting the number of cells that were both GFP- and mCherry+ from one image per well with 3 wells per condition (n = 3 separate experiments) using the Leica LAS X software. Images were assembled in Adobe Photoshop.

### Flow cytometry and FACS

For miRNA sensor analysis by flow cytometry, HER10 cells were transfected as described above. Immediately prior to analysis, cells were lifted from the plate with 0.25% Trypsin, washed with maintenance media, resuspended in 1 mL of FACS media (DMEM without phenol red supplemented with 1% FBS and 1 mM EDTA) containing Vybrant DyeCycle Violet DNA Stain (at 1 μl per 1 million cells; Molecular Probes) and incubated at 37 °C for 30 minutes. Cells were analyzed using a Becton Dickinson LSR-II flow cytometer. We analyzed 40,000 events per experiment (n = 3 experiments), and, on average, our gating strategy considered approximately 6,800 of these events as mCherry+, “transfected cells” to be used for downstream cell cycle analysis using the Watson (pragmatic) model in FlowJo (See Fig. S5 for gating strategy). We used the modeled cell cycle phase populations provided by FlowJo to analyze the level of sensor GFP in each phase of the cell cycle. When ClickIt EdU was used with flow cytometry (for let-7 overexpression and knockdown experiments or FACS-qPCR), cells were given a 30-min pulse of EdU (10 μM) and subsequently collected in Trypsin, as described above. After collection with Trypsin, cells were fixed for 5–10 min in 4% PFA at RT and stained using the Invitrogen ClickIt EdU 488 Detection Kit and stained with DAPI for >5 min. Cells were analyzed on a Beckman Coulter Cytoflex Flow Cytometer or sorted using a Beckman Coulter Astrios EQ Cell Sorter. Our initial gating strategy was similar to that described above, with adjustments highlighted in Fig. S5D. For flow cytometry in let-7 OE- and Ant-transfected cells, we measured the percentage of cells currently in S-phase by quantifying the number of cells in the 488+ population (EdU-incorporated). For FACS, cells were segregated into three populations based on their EdU/DAPI fluorescence (see Fig. S5D) with no fewer than 500k cells per population, and used for downstream analysis by qRT-PCR.

### miRNA qRT-PCR

Total RNA was extracted using the miRNeasy FFPE Kit (Qiagen) according to manufacturer’s protocol, with one exception: steps for paraffin removal were omitted. For miRNA RT, total RNA was reverse transcribed in multiplex using SuperSrcipt IV (Invitrogen) and custom designed stemloop reverse transcription primers similar to those previously described^29,70^ (primer sequences are listed in the Primer List). qPCR was performed on an Applied Biosystems StepOne qPCR system using SYBR green reagents (PowerSYBR Green PCR Master Mix; Applied Biosystems) and standard protocols.

### Live imaging

Her10 cells were plated on a 35 mm glass-bottom dish coated with basement membrane extract (BME, 1:60 dilution; Trevigen) and transfected with mCherry and sensor constructs as described above. The following day, 1 μM SiR-DNA (Cytoskeleton Inc.) and 10 μM Verapamil (Cytoskeleton Inc.) was added and cells were incubated at 37 °C for thirty minutes. For live imaging with FUCCI, the stable HER10-FUCCI cell line (described above) was transfected with control oligos, Let-7 mimic, or Let-7 Ant and were similarly incubated with SiR-DNA and Verapamil. Immediately after staining with SiR-DNA, we performed live imaging using an Andor Dragonfly spinning disk confocal microscope and Andor FUSION software. Images were captured every 7 minutes for 24–48hrs. Movies were edited and annotated in FIJI. Individual cell tracking and fluorescence quantification was performed using TrackMate (a plug-in provided in FIJI^71^). Statistical analysis was performed using the linear regression function in GraphPad Prism.

### *In utero* electroporation

In utero microinjection and electroporation was performed at E12 as described^69^. Briefly, timed-mated CD1 mice (Charles River Laboratories) were anesthetized and the uterine horns were exposed. 1 µl of DNA solution containing 0.5 µg/µl of pCAG-mCherry and 2 µg/µl of pCAG-Let-7 sensor or pCAG-miR1-sensor in 10 mM Tris (pH 8.0) and 0.01% Fast Green was injected per embryo. Forceps-type electrodes (Nepagene) with 5-mm pads were used for electroporation (five 50-msec pulses of 25 V). Electroporated embryos were collected 18–24 hours post-electroporation and processed for immunostaining.

### Sequential EdU/Ki67 labeling

HER10 cells, mCSCs or primary cultures were appropriately transfected (described above) using Lipofectamine 2000 (Invitrogen) according to manufacturer’s protocol. 24 hours after transfection, cells were given a 30-min pulse of 10 μM EdU (5-ethynyl-2’-deoxyuridine; Invitrogen). After EdU incubation, cells were washed 2X with PBS, new maintenance media was added, and cells were incubated for an additional 18 hours before fixation in 4% paraformaldehyde for 10 min at RT. After fixation, EdU was visualized using the ClickIT EdU Alexa Fluor 488 Plus imaging kit (Invitrogen) according to manufacturer’s protocol. After EdU visualization, samples were blocked with 4% non-fat dry milk in TST (10 mM Tris, 150 mM NaCl with 0.1% Tween) and immunostained overnight at 4 °C with rabbit anti-Ki67 (Clone SP6; Thermo Scientific) diluted 1:200 in blocking solution. After incubation with an Alexa Fluor 647-conjugated donkey-anti-rabbit secondary antibody (Invitrogen) diluted 1:200 in 1% non-fat dry milk in TST, cells were washed and imaged on a Leica DM 5000 M fluorescence microscope with a Leica DFC 500 camera. Quantitation was performed by counting the number of transfected (mCherry+) cells that were EdU+ and Ki67+ from 3–5 images per experiment (n = 3 separate experiments) using ImageJ. Images were assembled in Adobe Photoshop.

### Cumulative EdU labeling

Cumulative thymidine analog labeling is a well-established method for measuring cell cycle length within a population of cells^5,28^. Here, we cultured transfected HER10 cells or mCSCs and E11.5 mouse cortex primary cultures with a continuous supply of EdU (10 μM EdU, with media containing fresh 10 μM EdU added every 12 hours) for 24–50 hours. Samples were collected and fixed at 4-, 8-, 12-, 24- and 50-hour time points. After fixation, EdU was detected using the ClickIt EdU Detection kit (Invitrogen) and the proportion of EdU-positive cells over total DAPI-positive cells (HER10 cells) or Sox2 positive progenitors (cortex primary cultures). Cell cycle lengths were calculated by determining the X-intercept: of where the line generated from the 4-, 8- and 12-hour time points intersects with the growth fraction (the final percentage of cells that were EdU labeled in the end). At each time-point, we quantified 3 images per sample, with n = 3 samples per condition.

### Statistical analysis

Unless otherwise stated, statistical analysis was performed using GraphPad Prism and considered significant when P < 0.05. Statistical analysis was performed using either a student’s t-test or an Ordinary one-way ANOVA with Dunnett’s multiple comparisons, as appropriate. Data are presented as mean ± SEM. Normality was tested using either the D’Agostino & Pearson or Shapiro-Wilke tests in GraphPad Prism.

## Supplementary information

Supplementary information

Movie 5

Movie 4

Movie 3

Movie 2

Movie 1

## Data Availability

All data generated or analyzed during this study are included within this published article and its Supplementary Information files.
