# Author Correction: Let-7 regulates cell cycle dynamics in the developing cerebral cortex and retina

**DOI:** 10.1038/s41598-021-82224-1

**Published:** 2021-01-28

**Authors:** Corinne L. A. Fairchild, Simranjeet K. Cheema, Joanna Wong, Keiko Hino, Sergi Simó, Anna La Torre

**Affiliations:** Department of Cell Biology and Human Anatomy, University of California - Davis, Davis, CA USA

Correction to: *Scientific Reports* 10.1038/s41598-019-51703-x, published online 25 October 2019

This Article contains an error in Figure 5a, where the “S-phase Let-7s” data is duplicated for the “G2/M phase Let-7s” data in the middle graph of the top row.

The correct Figure 5a appears below as Figure 1.

**Figure 1:**
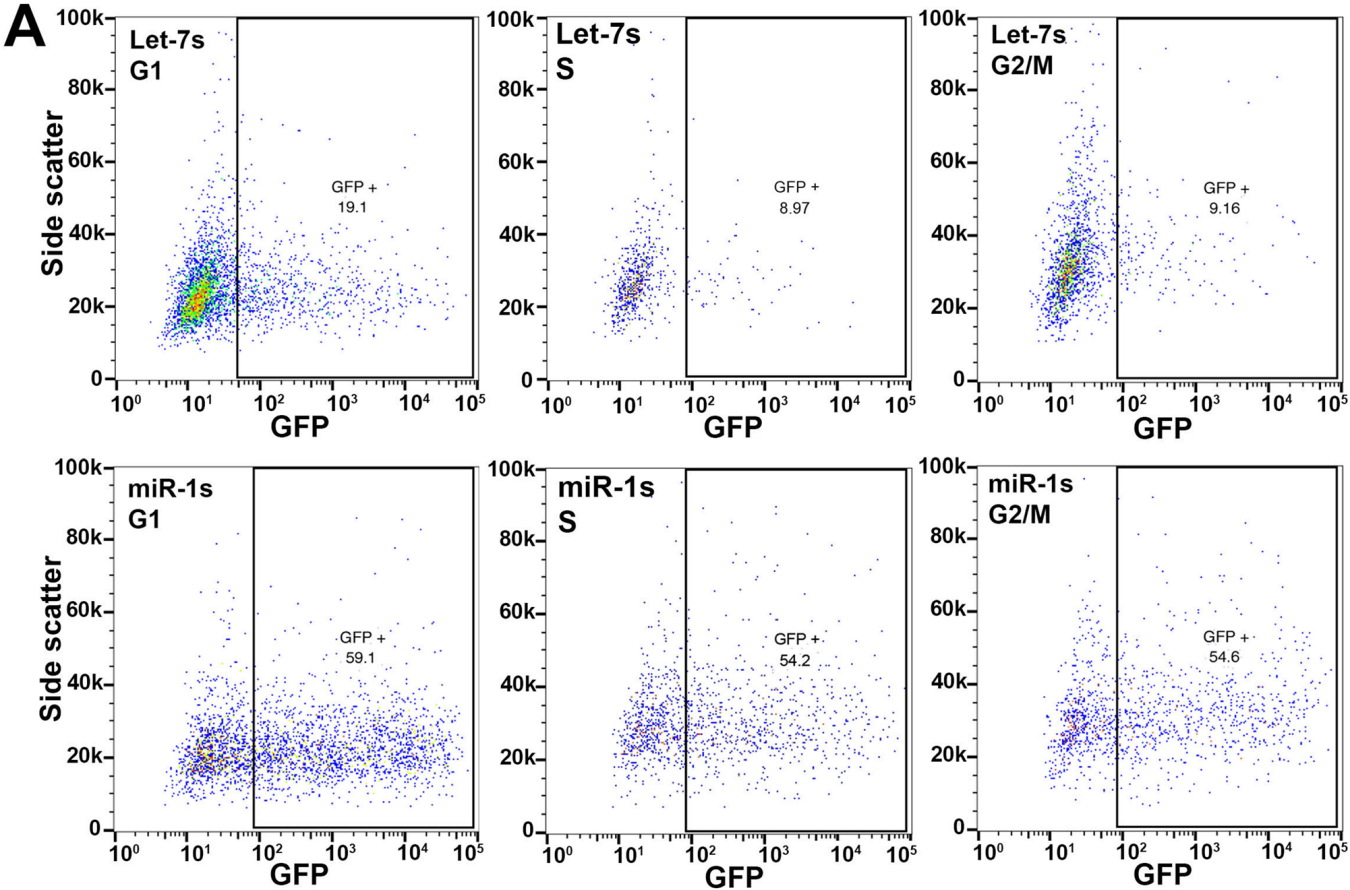
A correct version of the original Figure 5a.

