# Supplementary material for: Let-7 regulates cell cycle dynamics in the developing cerebral cortex and retina
Source: Sci Rep. 2019 Oct 25;9:15336. doi: 10.1038/s41598-019-51703-x (PMC6814839; doi:10.1038/s41598-019-51703-x)
Supplement: Supplementary file 1 — Supplementary information [file 41598_2019_51703_MOESM1_ESM.docx]

**Supplementary Information for:**

**Let-7 regulates cell cycle dynamics in the developing cerebral cortex and retina**

**Authors:** Corinne L. A. Fairchild, Simranjeet Cheema, Joanna Wong, Keiko Hino, Sergi Simo, and Anna La Torre

**Supplementary Figure 1**

**
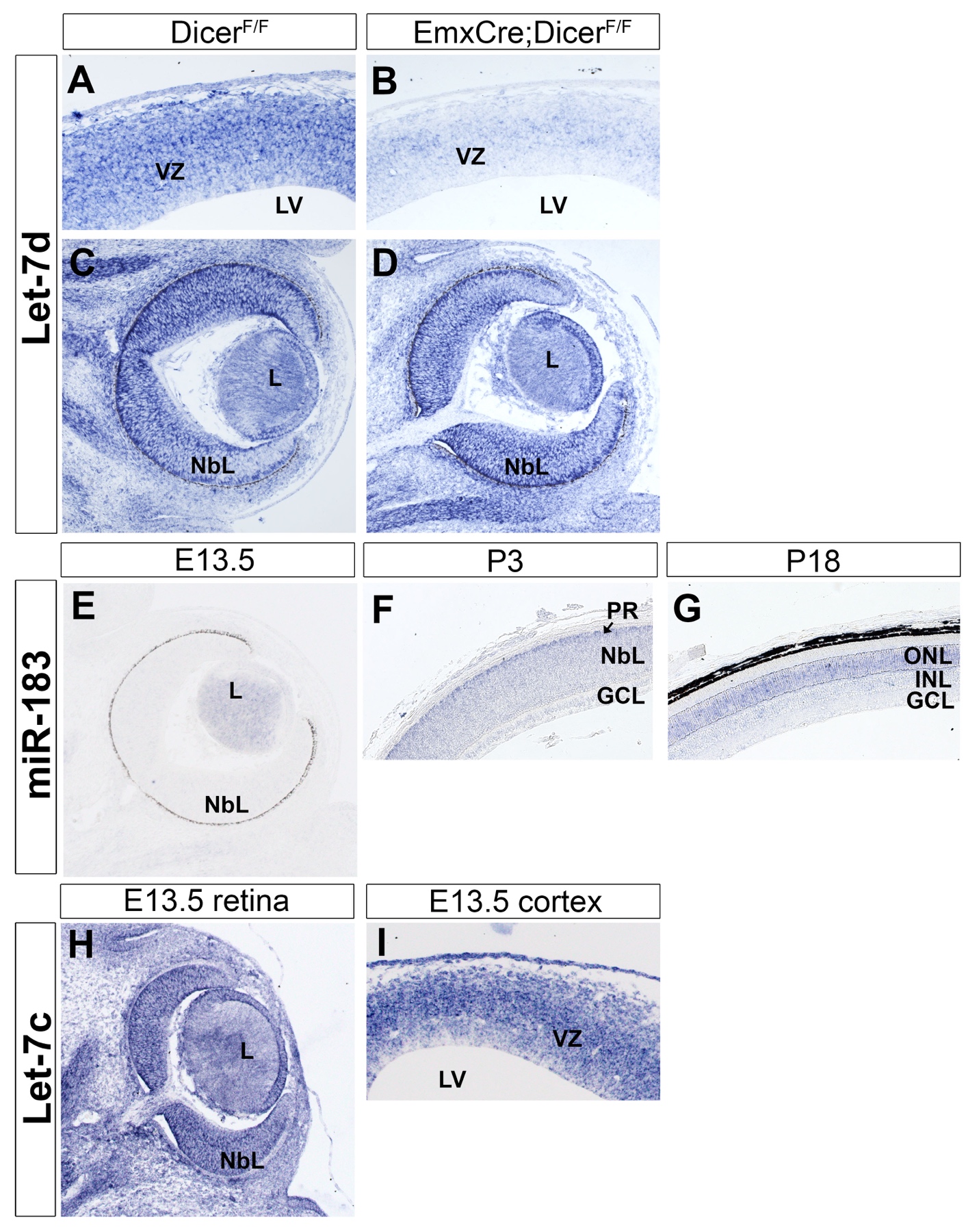
**

**Supplemental Fig. 1: Additional controls for let-7d *in situ* hybridization profile. (A-D)** *In situ* hybridization for let-7d in Dicer mutant animals. Let-7d expression at E13.5 is greatly reduced in the cortex Emx1-Cre;Dicer^F/F^ mutant animals (B) compared to wild-type littermates (A). As expected, let-7d levels in the retina, where Cre is not expressed, is comparable in both Emx1-Cre;Dicer^F/F^ mutants (C) and wildtype littermates (D) at E13.5. *In situs* shown here were performed simultaneously and NBT/BCIP development was stopped at the same time for all samples shown. **(E-F)** Expression profile for miR-183 in developmental and adult animals. Consistent with literature, we did not observe miR-183 expression in the retina at embryonic stages (E13.5, E). As early as P3, miR-183 expression can be observed in the immature photoreceptor layer (F). As expected, at P18, miR-183 expression was highly enriched in the ONL (G). **(H-I)** *In situ* hybridization for let-7c in E13.5 mouse retina (H) and cortex (I) shows that the pattern of let-7c expression greatly resembles that of let-7d at this stage (compare to Fig. 1). VZ, ventricular zone; LV, lateral ventricle; PR, photoreceptor layer; Nbl, neuroblastic layer; L, lens; GCL, ganglion cell layer; INL, inner nuclear layer; ONL, outer nuclear layer.

**Supplementary Figure 2**

**
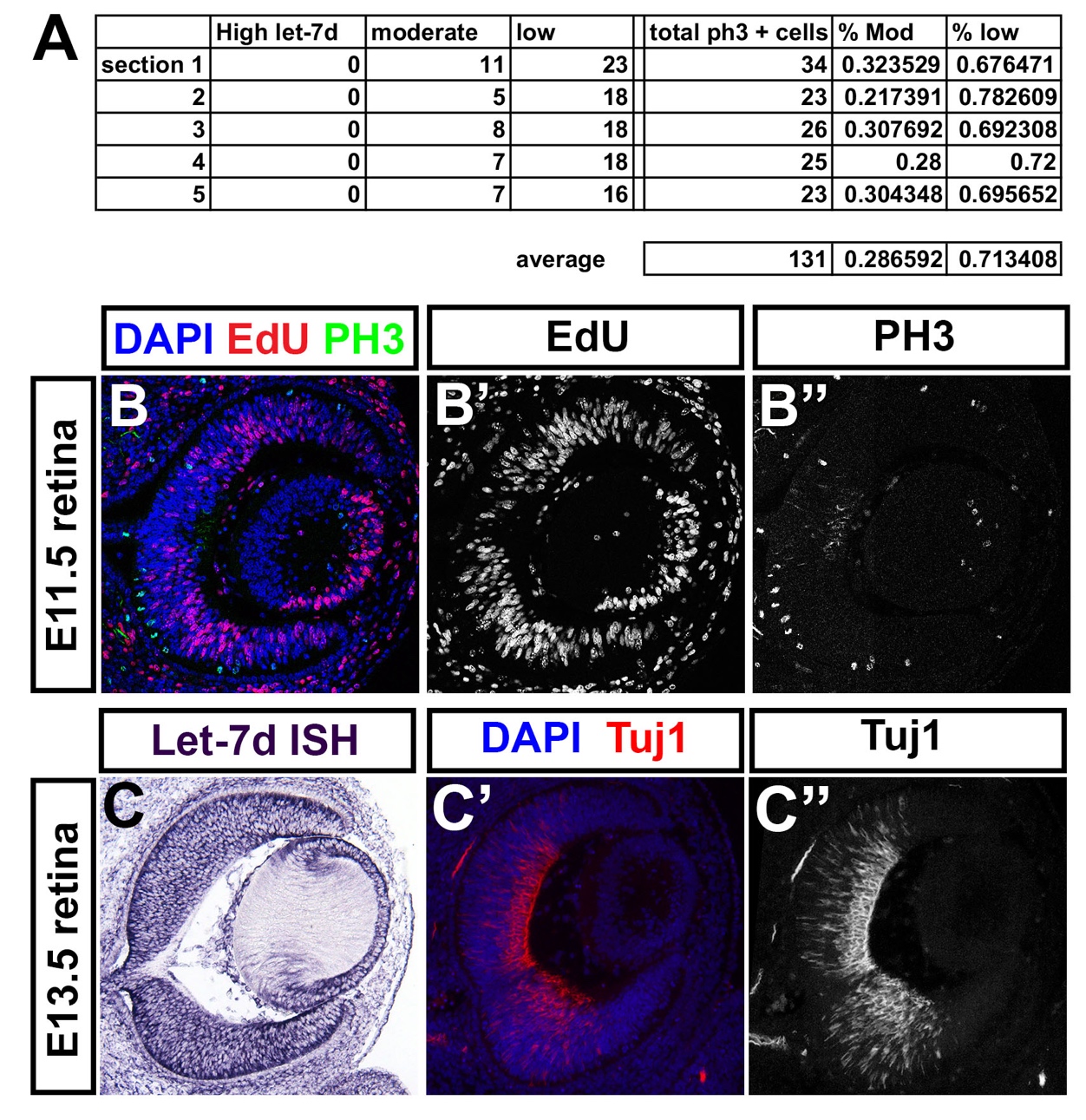
**

**Supplemental Fig. 2: Additional controls related to Figure 2. (A)** Quantification of the number of PH3+ cells that have low, moderate, or high levels of let-7d expression by *in situ* hybridization in the E13.5 mouse cortex. Over 5 sections from 2 animals (like those shown in Fig. 2C), we found that none of the PH3+ cells had high levels of let-7d, 28.7% of PH3+ cells had moderate levels of let-7d and 71.3% of PH3+ cells had low levels of let-7d. **(B)** E11.5 retina stained for DAPI (blue in B), EdU (red in B, white in B’) and PH3 (green in B, white in B”). These images are the same as those shown in Fig. 2J, but include separate channel images for EdU and PH3. **(C)** Example of one E13.5 retina stained by *in situ* hybridization for let-7d with side-by-side comparison of another section of E13.5 retina (same animal, different sections) stained with DAPI (blue in C’) and Tuj1 (red in C’, white in C”).

**Supplementary Figure 3**

**
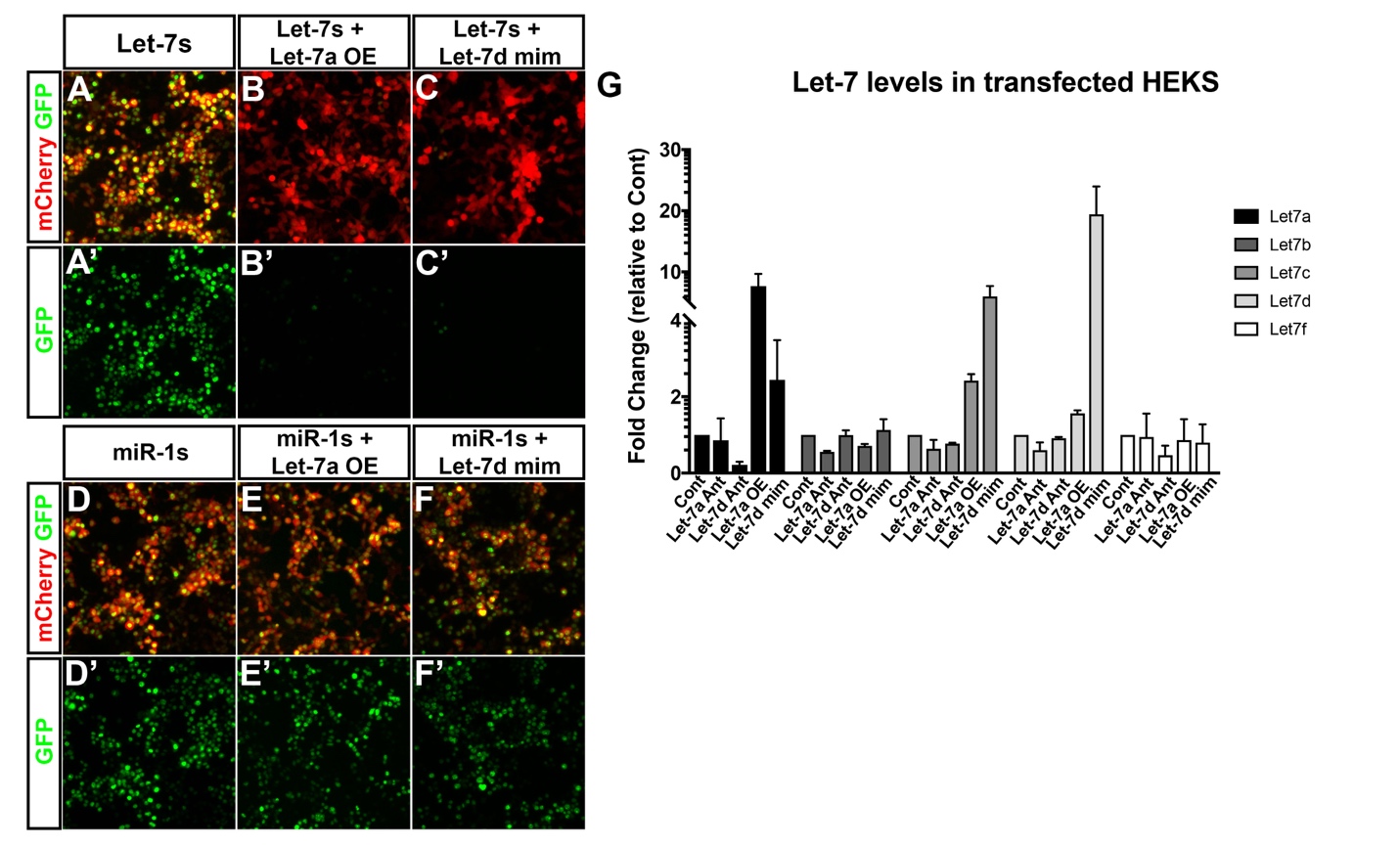
**

**Supplemental Fig. 3: Our tools are not specific for any one let-7 isoform. (A-F)** HEK293T cells transfected with Let-7s (A-C) or miR-1s (D-F). HEKs have very low endogenous let-7 activity, and thus nearly every transfected cell is GFP+ when let-7s is transfected with mCherry as a transfection control (green cells in A,A’). GFP from the sensor construct is repressed regardless of which let-7 isoform is present; for example, GFP is repressed when HEK293Ts are transfected with the let-7a OE DNA construct (compare GFP+ cells in A’ and B’) or a let-7d mimic (compare GFP+ cells in A’ and C’). As expected, GFP expression in miR-1s transfected HEKs was not affected by transfection with the let-7a OE DNA construct (compare GFP+ cells in D’ and E’) or a let-7d mimic (compare GFP+ cells in D’ and F’). **(G)** qRT-PCR for a variety of let-7 isoforms (from left to right: let-7a (black bars), let-7b (dark grey bars), let-7c (grey bars), let-7d (light grey bars), and let-7f (white bars)) in HEK293T cells after let-7 was knocked down using a let-7a or let-7d AntagomiR or overexpressed using the let-7a OE DNA construct or let-7d mimic. These results show that our let-7 PCR primers are not specific for one isoform, and could assay changes in the level of other let-7 family members.

**Supplementary Figure 4
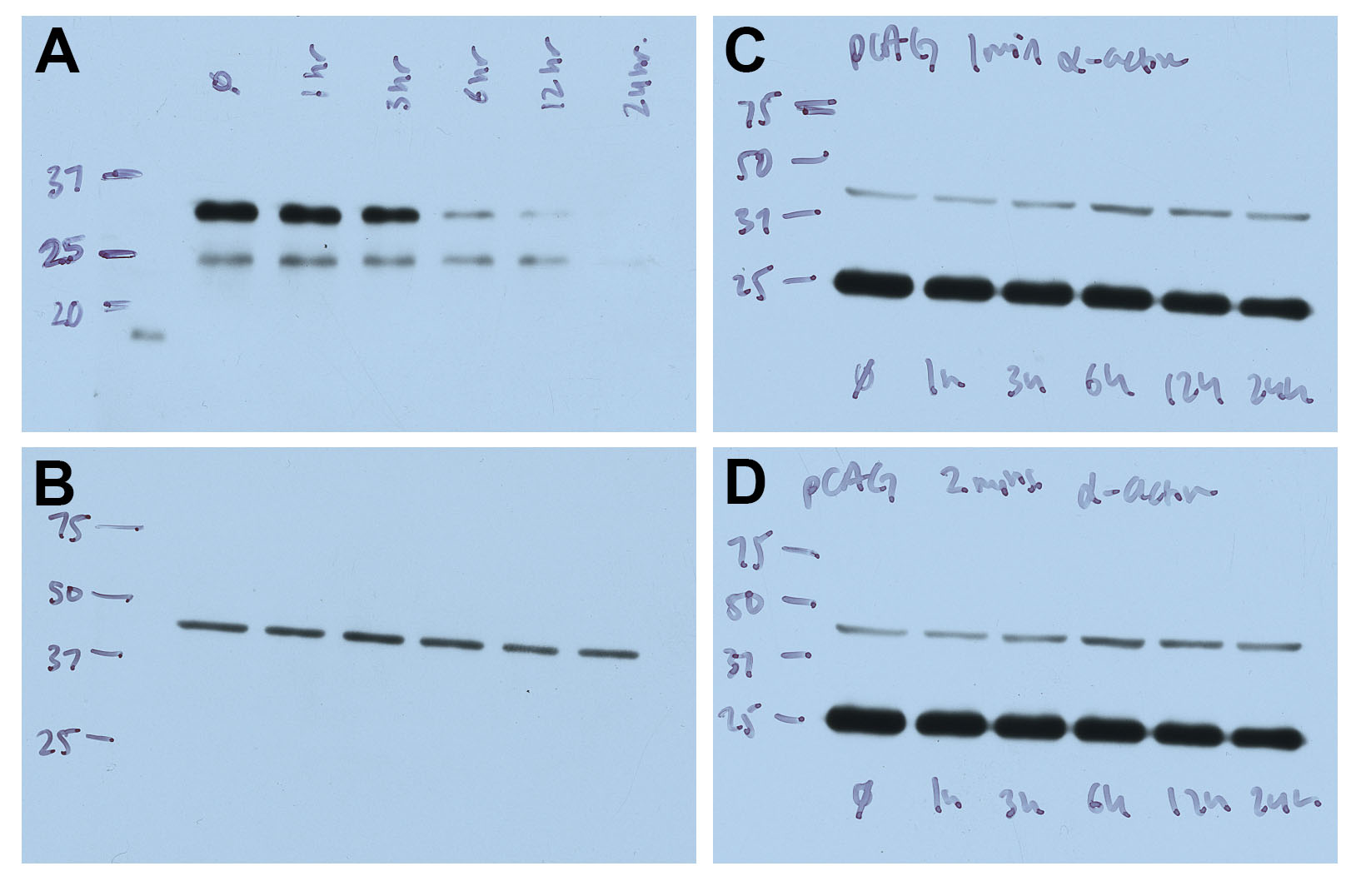
**

**Supplemental Fig. 4:** **Unaltered images of Western Blot Analysis from Figure 3.**  **(A)** HER10 cells transfected with Let-7s and collected 0, 1, 3, 6, 12, or 24 hours after beginning cyclohexamide treatment. Western blotting was performed using a rabbit anti-GFP antibody (Invitrogen). Top band corresponds to GFP from the Let-7s. Bottom band is a non-specific band. An image of the top set of bands is shown in the top right panel of Fig. 3F (AcGFP). **(B)** The same lysates from (A) were run on a separate gel and western blot was performed using an anti-Actin antibody. This image was shown in the bottom right panel of Fig. 3F (actin). **(C)** HER10 cells transfected with pCAG-eGFP and collected 0, 1, 3, 6, 12 or 24 hours after beginning cyclohexamide treatment. Western blotting was performed using the same rabbit anti-GFP antibody in A (bottom band). The membrane was then re-probed, without stripping, for actin (top band). The bottom set of bands (which correspond to eGFP) were used in the top left panel in Fig. 3F. **(D)** Longer exposure of the blot shown in (C). The top set of bands (corresponding to Actin) were used in the bottom left panel in Fig. 3F.

**Supplementary Figure 5**

**
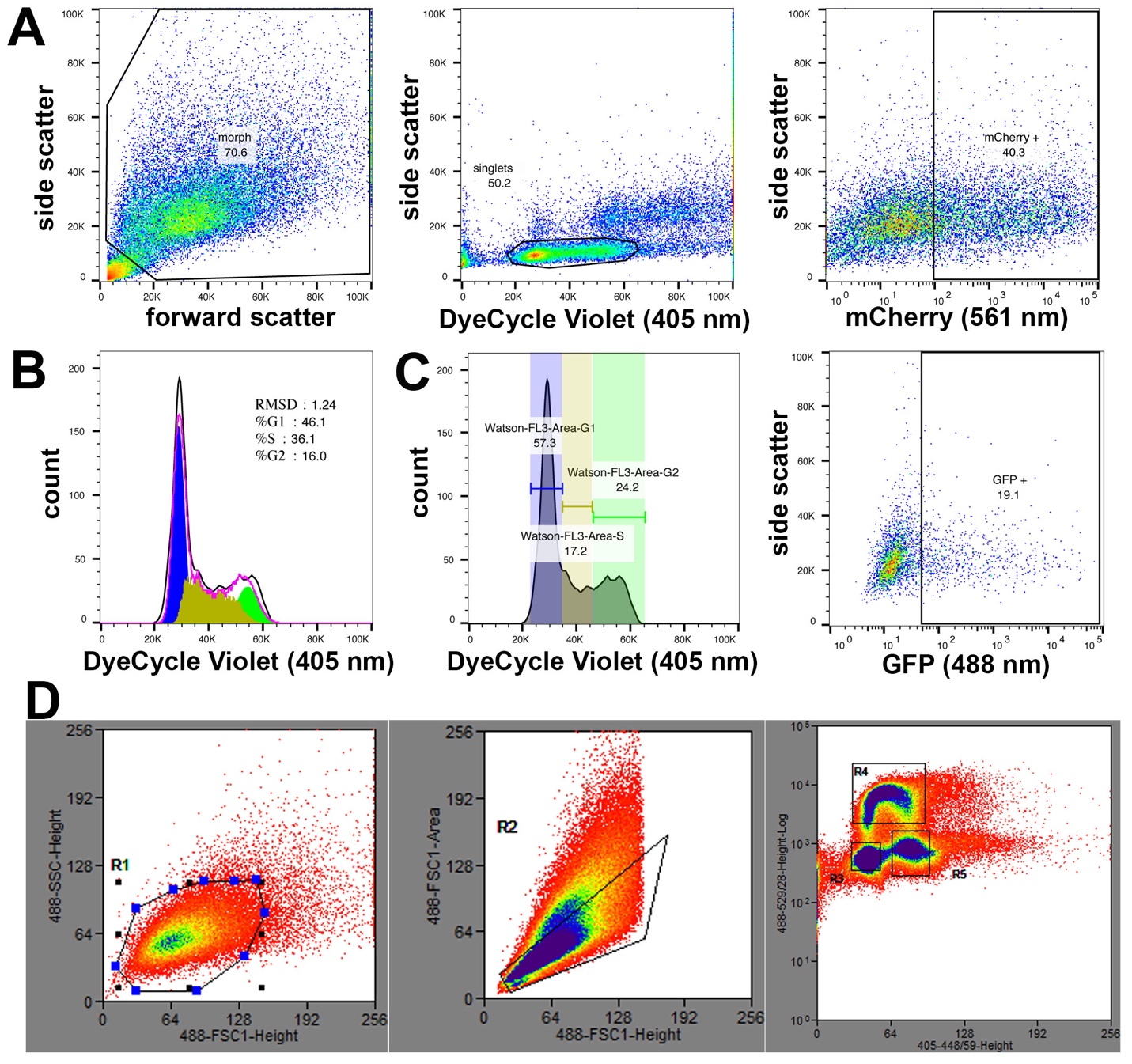
**

**Supplemental Fig. 5: Gating strategy for cell cycle analysis by flow cytometry.** **(A)** To assess cell cycle phase, HER10 cells were transfected with mCherry and the miRNA sensor (containing GFP) and stained with DyeCycle Violet (DCV). The sample was first analyzed for proper morphology using side- and forward-scatter plots (left panel). We then performed a doublet discrimination by plotting side-scatter and DCV fluorescence to ensure only singlets were analyzed (middle panel). Lastly, we excluded non-transfected cells by gating for cells that were mCherry-positive (right panel). **(B)** Following the gating scheme in (A), DCV histograms were analyzed to determine the number of cells in each phase of the cell cycle using the Watson (Pragmatic) model in FlowJo. A representative example of this analysis is shown in (B). The percent of cells in each phase of the cell cycle is shown, along with the RMSD, which is a measure of how well the data fits the model. RMSD values ranged from 0.67-3.39 for all experiments. **(C)** For miRNA sensor activity experiments (shown in Fig. 5A,B), we gated each phase of the cell cycle using the model in) (B; see left panel in C and analyzed the percentage of cells that were GFP-positive in each of these populations. A representative example is shown (right panel). **(D)** Representative example of the gating strategy for FACS-qPCR (Fig. 5C) and S-phase flow cytometry experiments (Fig. 7G) using ClickIt and DAPI staining. Morphology and doublet discrimination gating was performed as usual (D, left two panels; see above) and, if applicable, cells were gated for mCherry (for transfected cells, Fig. 7). Cells were analyzed by plotting GFP (log scale, Y-axis) and DAPI (linear scale, X-axis) fluorescence, and segregated into three populations G1 (1X DAPI, GFP-negative; R3 population box in D, right panel), S (GFP+; R4 population box in D, right panel), and G2/M (2X DAPI, GFP-negative; R5 population box in D, right panel).

**Supplemental Figure 6**


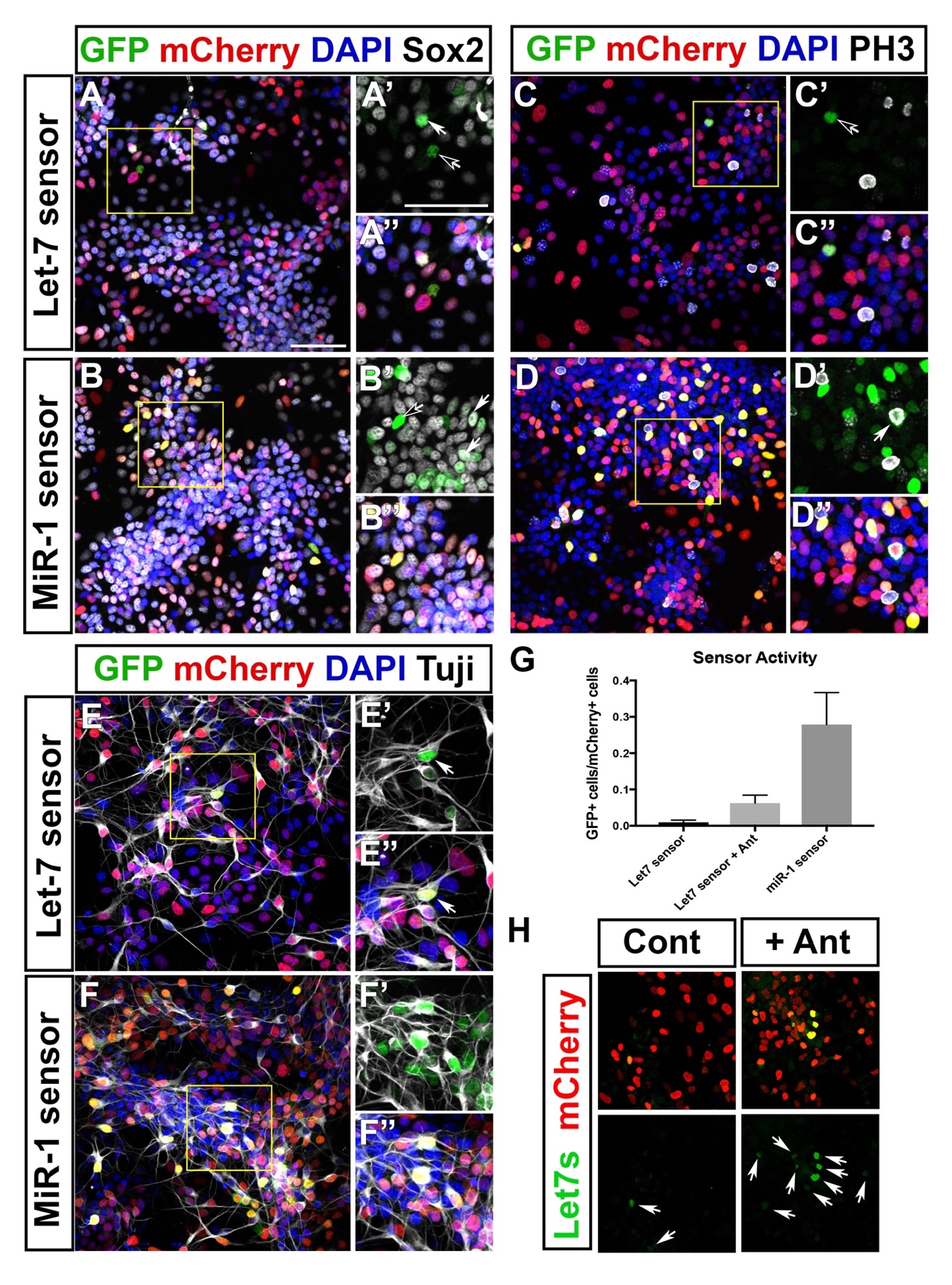


**Supplemental Fig. 6: E11.5 mouse cortex primary cultures.** miRNA activity in E11.5 mouse cortex primary cultures by let-7 and miR-1 sensor analysis and immunostaining. **(A,B)** E11.5 mouse cortex primary cultures transfected with either let-7s (A) or miR-1s (B) and stained for Sox2. Compared to miR-1 sensor transfected primary cultures, very few let-7 sensor transfected cells had detectable levels of GFP, suggesting that let-7 activity is endogenously high in these cultures. The majority of GFP+ cells in let-7 sensor transfected primary cultures were not Sox2+ cortical progenitors (black arrow in A’); one rare example is shown by white arrow in A’. **(C,D)** E11.5 mouse cortex primary cultures transfected with either let-7s (C) or miR-1s (D) and stained for PH3. Although there were some GFP+ and PH3+ cells in miR-1 sensor transfected cultures (white arrow in D’), we did not observe any GFP+ and PH3+ cells in let-7 sensor transfected cultures (black arrow in C’). **(E,F)** E11.5 mouse cortex primary cultures transfected with either let-7s (E) or miR-1s (F) and stained for Tuj1. Many of GFP+ cells in both let-7 and miR-1 sensor transfected cultures were also positive for the neuronal marker Tuj1, suggesting that the few GFP+ cells we observe in our let-7 sensor transfected primary cultures are the likely result of a subset of post-mitotic neurons that down regulate let-7 upon differentiation. **(G)** Quantification of the proportion GFP+ cells in cultures transfected with Let-7s alone, Let-7s with Let-7 Ant and miR-1s. Co-transfection of let-7s with antagomiR can increase sensor GFP, suggesting that our antagomiR is effective in our culture system. **(H)** Representative example of the data shown in G. Scale bars in (A)= 50 μm.

**Supplemental Figure 7**


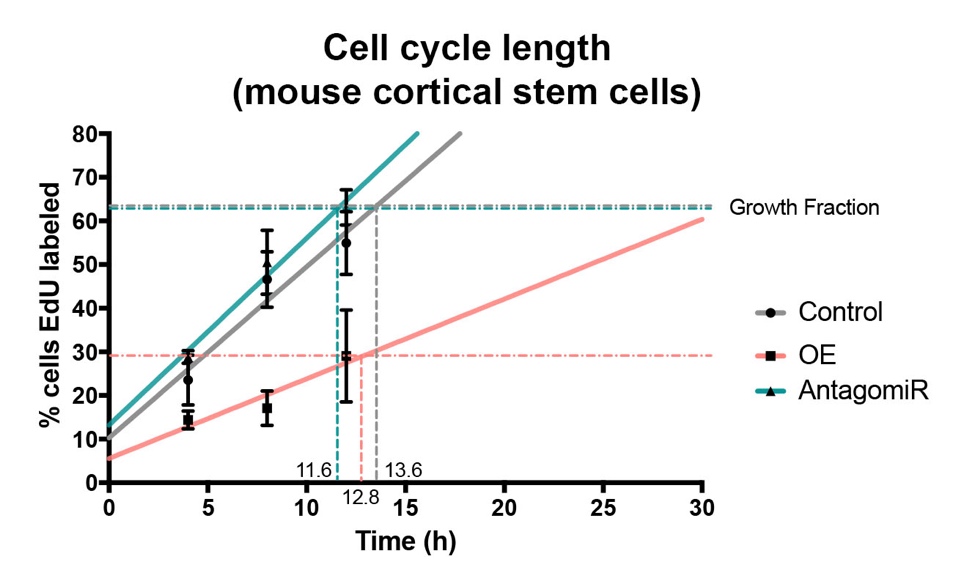


**Supplemental Fig. 7:** **Cumulative EdU labeling in mCSCs.** mCSCs were transfected with mCherry alone (Cont; grey line), a let-7d mimic (OE; orange line) or a let-7 antagomiR (Ant; teal line). The percentage of EdU-positive cells was visually quantified at 4-, 8-, 12-, and 24-hour time points (after addition of EdU) n=3 samples per condition. Growth fraction = the percent of EdU-positive cells at the final time point. The data shown here are the same as in Fig. 8, but include cells transfected with the let-7 mimic (Let-7 OE).
